# Prediction of intrinsic two-dimensional ferroelectrics in In_2_Se_3_ and other III_2_-VI_3_ van der Waals materials

**DOI:** 10.1038/ncomms14956

**Published:** 2017-04-07

**Authors:** Wenjun Ding, Jianbao Zhu, Zhe Wang, Yanfei Gao, Di Xiao, Yi Gu, Zhenyu Zhang, Wenguang Zhu

**Affiliations:** 1 Department of Physics, University of Science and Technology of China, Hefei, Anhui 230026, China; 2 International Center for Quantum Design of Functional Materials (ICQD), Hefei National Laboratory for Physical Sciences at the Microscale, and Synergetic Innovation Center of Quantum Information and Quantum Physics, University of Science and Technology of China, Hefei, Anhui 230026, China; 3 Key Laboratory of Strongly-Coupled Quantum Matter Physics, Chinese Academy of Sciences, School of Physical Sciences, University of Science and Technology of China, Hefei, Anhui 230026, China; 4 Beijing Computational Science Research Center, Beijing 100084, China; 5 Department of Materials Science and Engineering, University of Tennessee, Knoxville, Tennessee 37996, USA; 6 Materials Science and Technology Division, Oak Ridge National Laboratory, Oak Ridge, Tennessee 37831, USA; 7 Department of Physics, Carnegie Mellon University, Pittsburgh, Pennsylvania 15213, USA; 8 Department of Physics and Astronomy, Washington State University, Pullman, Washington 99164, USA

## Abstract

Interest in two-dimensional (2D) van der Waals materials has grown rapidly across multiple scientific and engineering disciplines in recent years. However, ferroelectricity, the presence of a spontaneous electric polarization, which is important in many practical applications, has rarely been reported in such materials so far. Here we employ first-principles calculations to discover a branch of the 2D materials family, based on In_2_Se_3_ and other III_2_-VI_3_ van der Waals materials, that exhibits room-temperature ferroelectricity with reversible spontaneous electric polarization in both out-of-plane and in-plane orientations. The device potential of these 2D ferroelectric materials is further demonstrated using the examples of van der Waals heterostructures of In_2_Se_3_/graphene, exhibiting a tunable Schottky barrier, and In_2_Se_3_/WSe_2_, showing a significant band gap reduction in the combined system. These findings promise to substantially broaden the tunability of van der Waals heterostructures for a wide range of applications.

## Abstract (web summary)

The development of devices based on 2D materials beyond graphene benefits from identifying compounds with diverse functional properties. Here, the authors predict computationally that 2D In_2_Se_3_ and related materials are room temperature ferroelectrics with both in- and out-of-plane polarization.

Ferroelectricity, a property of materials associated with the emergence of spontaneous electric polarization, has a wide range of technological applications, such as non-volatile memories, field effect transistors and sensors^1^,^2^. Previous studies of ferroelectric materials have mainly focused on complex oxides, such as ABO_3_ perovskite compounds^3^. Driven by technological demand for device miniaturization, exploration of the ferroelectric properties of perovskite thin films has been made more intensively^1^,^4^,^5^. Separately, a rapidly increasing number of two-dimensional (2D) van der Waals materials have been discovered, exhibiting a rich variety of emergent physical properties^6^,^7^,^8^,^9^,^10^. These developments in principle may offer new and alternative opportunities for realizing ferroelectricity in the ultimate single-layer regime^11^,^12^, especially with regard to the most technologically relevant polarizability perpendicular to the film direction.

The existence of 2D ferroelectricity was predicted long time ago based on an idealized Ising model^13^, but realistic materials normally suffer from the fundamental constraint that ferroelectricity would disappear when the film thickness is below a critical value, due to the effects of surface energy, depolarizing electrostatic field and electron screening^4^,^5^,^14^,^15^,^16^. In general, the emergence of electric polarization demands breaking of the structural centrosymmetry in the polarization direction. Yet in the pristine structures of all known 2D materials including the well-known graphene and transition-metal dichalcogenides, the projections of their atomic positions on the out-of-plane axis are exclusively centrosymmetric, seemingly excluding any possible out-of-plane polarization.

Here we present the discovery of a class of stable single-layer 2D ferroelectric materials based on III–VI compounds in the form of III_2_–VI_3_. Using first-principles density-function theory (DFT) calculations, we reveal that the ground state structures of an intrinsic prototypical In_2_Se_3_ quintuple layer (QL) possess both spontaneous out-of-plane and in-plane electric polarization, which can be reversed via laterally shifting the central Se layer through readily accessible kinetic pathways with the assistance of a modest out-of-plane or in-plane electric field. Furthermore, we demonstrate tunability in the Schottky barrier height within an In_2_Se_3_/graphene junction and a significant band gap reduction in the van der Waals heterostructure of In_2_Se_3_/WSe_2_, in each case achieved by reversing the out-of-plane polarity of the In_2_Se_3_ layer. These findings effectively classify the well-known III_2_–VI_3_ compounds actually as the long-sought 2D ferroelectric materials.

## Results

### Structures of In_2_Se_3_ layered phases

**Figure 1:**
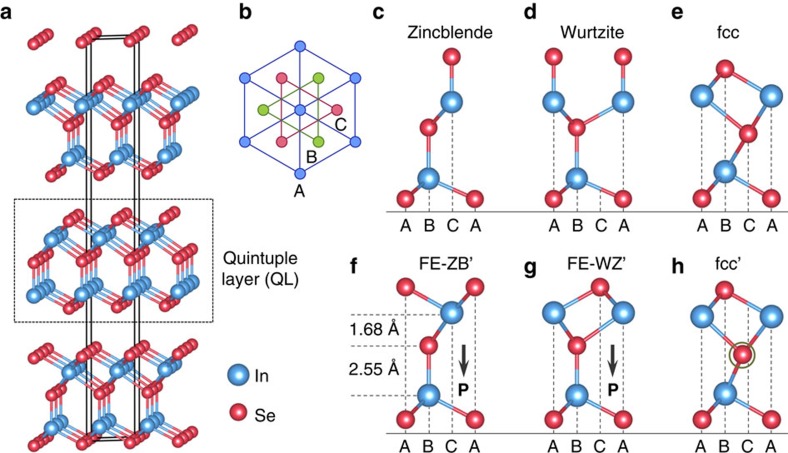
Layered structures of In_2_Se_3_. (**a**) Three-dimensional crystal structure of layered In_2_Se_3_, with the In atoms in blue and Se atoms in red, and a quintuple layer (QL) is indicated by the black dashed square. (**b**) Top view of the system along the vertical direction. Each atomic layer in a QL contains only one elemental species, with the atoms arranged in one of the triangular lattices A, B or C as illustrated. (**c**–**h**) Side views of several representative structures of one QL In_2_Se_3_, among which the **c**–**e** structures are derived from the zincblende, wurtzite and fcc crystals, respectively. In **f**, the interlayer spacings between the central Se layer and the two neighbouring In layers are displayed. The black arrows in **f**,**g** indicate the directions of the spontaneous electric polarization (P) in the FE-ZB′ and FE-WZ′ structures, respectively.

Before exploring the ferroelectric properties, we first systematically examine the detailed structures of In_2_Se_3_. Bulk In_2_Se_3_ has been shown to exist in two layered crystalline phases named *α* and *β* (refs 17, 18), formed by vertical stacking of two different types of covalently bonded 2D In_2_Se_3_ QLs via weak van der Waals interactions (Fig. 1a,b). The van der Waals nature of the inter-QL force is supported by earlier experimental observations that few-layer In_2_Se_3_ samples can be obtained by exfoliation^19^,^20^ or chemical vapour deposition^21^. Each atomic layer in a QL contains only one elemental species, with the atoms in a given layer arranged in a triangular lattice. The five atomic layers in a QL then stack in the sequence of Se-In-Se-In-Se atomic layers. Despite extensive experimental studies of the bulk structures, the precise alignments of the atomic layers within the *α* and *β* phases are still controversial^17^,^18^,^19^,^21^,^22^,^23^.

In this study, as prerequisites we employed DFT calculations to explore all possible atomic configurations (Fig. 1c–h and Supplementary Fig. 1) within a QL, including the ones derived from the most-common crystal structures, such as zincblende, wurtzite and face-centred cubic (fcc). From the calculated total energy versus lattice constant for each structure (Supplementary Fig. 2), we find that none of the zincblende, wurtzite or fcc structures is stable. In either the zincblende (ABBCC) or wurtzite (ABBAA) structure shown in Fig. 1c,d, the top of the QL terminates with a Se layer sitting on top of a second In layer, with each Se atop atom forming a single Se–In covalent bond. These high-energy structures can be substantially stabilized when the Se atoms in the top layer execute a lateral structural collective shift, leading to two energetically degenerate ground state structures, FE-ZB′ (ABBCA) and FE-WZ′ (ABBAC) (Fig. 1f,g), with the total heights of one QL around 6.8 Å. The dynamical and thermal stability of each of the two structures is further examined by calculating the phonon band structures (Supplementary Fig. 4a,b), confirming the absence of imaginary phonon modes, and *ab initio* molecular dynamic simulations (Supplementary Fig. 5). In addition, we find that the highly symmetric fcc (ABCAB) structure (Fig. 1e) is unstable, and a metastable structure, fcc′, can be derived by shifting the central Se layer slightly away from the ideal fcc positions (Fig. 1h). The total energy of this metastable fcc′ structure is 0.057 eV per unit cell higher than the two degenerate ground state structures. The most stable FE-ZB′ and FE-WZ′ and the metastable fcc′ structures are all semiconductors. Their calculated band structures are provided in Supplementary Fig. 6.

Based on the structural results presented above, we also obtain that the two degenerate ground states of FE-ZB′ and FE-WZ′ have an in-plane lattice constant of 4.106 and 4.108 Å, respectively, while the metastable state of fcc′ has an in-plane lattice constant of 4.048 Å. When compared with the experimentally measured lattice constants, we identify the ground states to be the *α* phase, while the fcc′ state to be the *β* phase^19^,^22^. Furthermore, our detailed calculations confirm that the experimentally observed Raman active A1 mode undergoes a blue shift when the In_2_Se_3_ structure transforms from the *α* phase to the *β* phase (Supplementary Fig. 4)^19^,^24^. Additionally, we note that the metastable nature of the *β* phase is consistent with the experimental observation that it is reached at higher temperature from the *α* phase through a structural phase transition^17^,^25^. In contrast, in a related recent DFT study, only one ground state was considered for the *α* phase, while the energetically higher fcc phase was identified to be the *β* phase^26^.

### Ferroelectric nature of In_2_Se_3_

**Figure 2:**
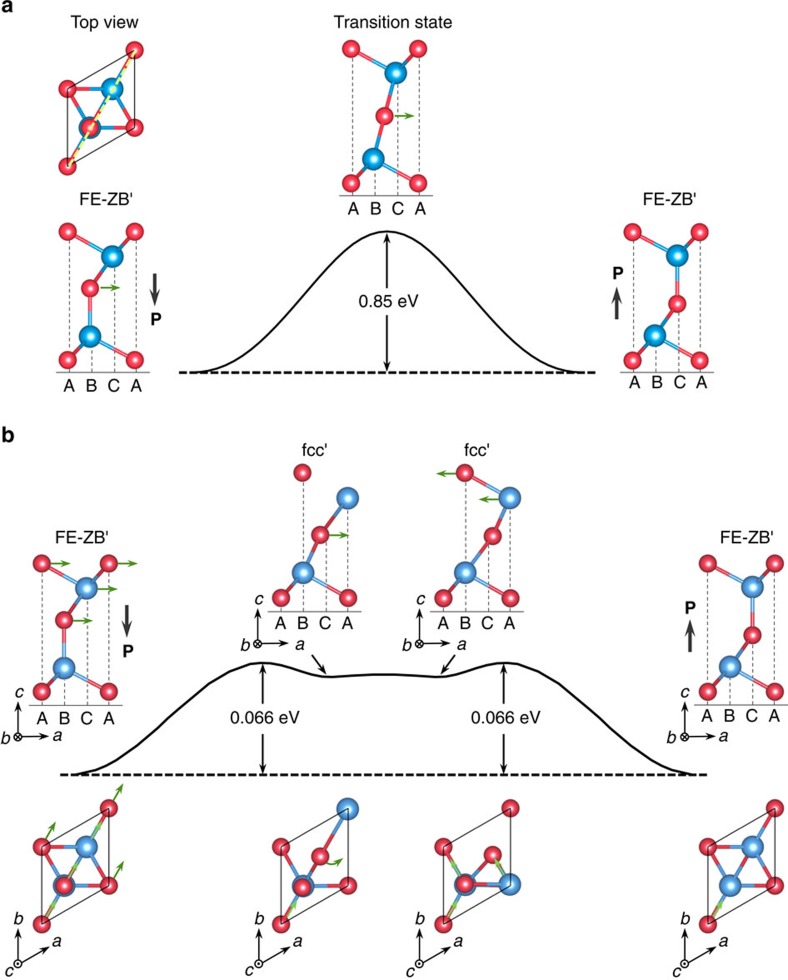
Kinetics pathways of polarization reversal processes. (**a**) Evolution of the total energy (*y* axis) of 1 quintuple layer (QL) In_2_Se_3_ in the FE-ZB′ phase transforming from the state with the electric polarization pointing downward (left) to the state with the electric polarization pointing upward (right) via a direct shifting process: the Se atoms in the central layer laterally shift from the B to C sites. The green arrows attached to atoms indicate the directions of atomic motion during the polarization reversal processes, which are in the plane perpendicular to the In_2_Se_3_ layer and passing through the green dashed line as shown in the top view. (**b**) Energy profile of the most effective kinetic pathway to reverse the orientation of the electric polarization of one QL In_2_Se_3_ in the FE-ZB′ phase involving a three-step concerted mechanism, as detailed in the main text. The activation barrier of the concerted motion is lower than the direct shifting process by an order of magnitude (note that the height of the barrier shown in **a** is scaled down by a factor of 10).

**Figure 3:**
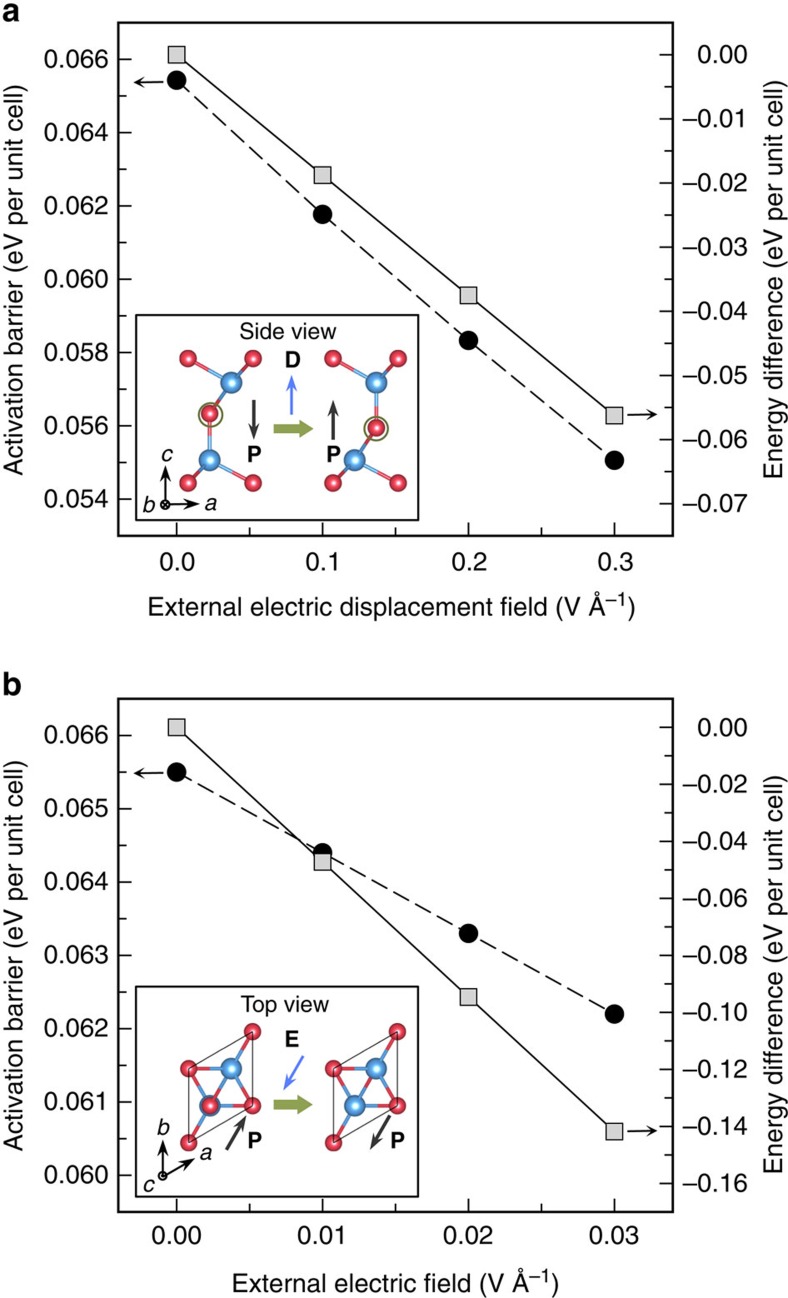
Effects of external electric fields. The calculated activation barrier (black circles) and energy difference (grey squares) between the initial and final states (the insets) in the electric polarization reversal process of 1 quintuple layer In_2_Se_3_ via the concerted motion as illustrated in Fig. 2b, plotted as a function of the external electric field applied in the out-of-plane direction (**a**) and in-plane [110] direction (**b**), respectively. The directions of the applied external electric fields are indicated by the blue arrows in the insets.

Next, we turn to the key prediction that each of the degenerate ground-state structures of the In_2_Se_3_ QL is an intrinsic 2D ferroelectric material with both out-of-plane and in-plane electric polarization. As shown in Fig. 1f,g, the Se atoms in both the top and the bottom surface layers reside on the hollow sites of the respective second-layer In atoms, while each atom in the central Se layer is tetrahedrally coordinated by the two neighbouring In layers, with one Se–In bond connecting to one side vertically and three Se–In bonds to the other side. As a result, the interlayer spacing between the central Se layer and the two In layers is dramatically different, effectively breaking the centrosymmetry and providing the very underlying basis for the emergence of the spontaneous out-of-plane electric polarization. The calculated magnitudes of the electric dipoles for one QL In_2_Se_3_ in the degenerate ground states are both around 0.11 eÅ per unit cell (calculated by HSE06, 0.094 eÅ per unit cell by generalized gradient approximation-Perdew-Burke-Ernzerhof (GGA-PBE)). In addition, each ground-state structure hosts two equivalent states with opposite electric polarizations, which only differ by the energetically degenerate positions of the central Se-layer atoms. Specifically, in the FE-ZB′ structure illustrated in Fig. 2a, the atoms in the central Se layer are at the B sites vertically aligned with the lower In layer (left in Fig. 2a), and the resultant electric dipole points downwards; by moving the central Se layer to the neighbouring C sites aligned with the upper In layer (right in Fig. 2a), the resultant electric dipole points upwards. In addition, the *α*-phase FE-ZB′ and FE-WZ′ structures also have in-plane electric polarization due to the in-plane centrosymmetry breaking. The in-plane electric polarization is along the [110] direction defined by the lattice vectors as illustrated in the insets of Fig. 3b. The magnitude of the in-plane electric polarization is calculated to be 2.36 and 7.13 eÅ per unit cell for the FE-ZB′ and FE-WZ′ phases, respectively, using the Berry phase approach. This difference can be attributed to the ions that make the two structures deviate from the non-polar reference structure possessing different numbers of charge, as illustrated in Supplementary Fig. 10a.

A critical issue for ultrathin ferroelectric materials is the depolarization effect. It is known that the ferroelectricity of conventional ferroelectric thin films is usually suppressed, as the films are thinner than a critical thickness due to the effects of a depolarizing field induced by uncompensated charges in the presence of metal electrodes^4^,^5^,^14^,^15^. To examine the influence of the depolarizing field on the stability of the ferroelectric phase of In_2_Se_3_, we performed calculations with supercells containing one QL of In_2_Se_3_ sandwiched between two graphite electrodes in short-circuit, as illustrated in Supplementary Fig. 7a,b for the In_2_Se_3_ layer in the ferroelectric FE-ZB′ phase and non-polar fcc′ phase, respectively. A detailed description of the calculations is provided in Supplementary Note 1. The calculated results confirm that the depolarization effects only slightly reduce the energy difference between the two phases of In_2_Se_3_, and the FE-ZB′ phase is still more stable than the non-polar fcc′ phase. The electrostatic potential plot, shown in Supplementary Fig. 7c, also indicates that the built-in electric field within the ferroelectric In_2_Se_3_ layer still exists in the presence of the graphite electrodes. All these results predict a realistic material system that is energetically stable and possesses 2D ferroelectricity with out-of-plane electric polarization at the single-layer limit.

For multilayer In_2_Se_3_ films, their net polarization increases as a function of the film thickness and saturates as the thickness is above two QLs, in the cases that the polarization of all the ferroelectric QLs is aligned along the same orientation at each thickness. Detailed calculation results and discussions are provided in Supplementary Fig. 8 and Supplementary Note 2.

It is known that all ferroelectric materials are also piezoelectric and pyroelectric. We have investigated the piezoelectric property of a single QL of ferroelectric In_2_Se_3_ by calculating the variation of the electric dipole as a function of the in-plane lattice deformation (Supplementary Fig. 9a) and the height variation as a function of an external electric displacement field applied in the vertical direction (Supplementary Fig. 9b). The results indicate that the compressive strain has a more significant effect on the electric polarization than the tensile strain. In particular, a tensile strain can slightly enhance the dipole moment of the ferroelectric layer. Furthermore, the electric-field-induced height variation in one QL ferroelectric In_2_Se_3_ is very small, on the order of 10^−3^ Å at the electric displacement field as large as 0.2 V Å^−1^, which would be challenging to be resolved by piezoresponse force microscopy.

### Kinetics of polarization reversal

To further demonstrate the ferroelectric nature of the systems, we must show that the direction of the electric polarization can be readily reversed by the application of a physically realistic electric field. We address this issue in two stages: first, we identify the most effective kinetic pathway connecting the two degenerates states with different polarities in the absence of an electric field; secondly, we investigate the effect of an external electric field on further reduction of the activation barrier along the pathway.

As a brute force check in the first stage, we find that the activation barrier against direct shifting of the central Se layer is 0.85 eV per unit cell, as shown in Fig. 2a. More importantly, an alternative process with a significantly lower activation barrier to reverse the electric polarization is revealed via a three-step concerted motion of the upper three Se-In-Se layers, as illustrated in Fig. 2b. In the first step, the *α*-phase FE-ZB′ structure transforms into the metastable *β*-phase fcc′ structure by laterally shifting the top three atomic layers together along the same direction to neighbouring sites. In the second step, the central Se atoms rotates around the C sites by 60° to a degenerate fcc′ structure. In the third step, only the top two layers laterally shift along a direction that is rotated away from the original shifting direction by 60°, finally reaching an equivalent FE-ZB′ structure, but now with the electric polarization reversed. The overall activation barrier of this concerted process is much lower, with the highest barrier to be only 0.066 eV per unit cell along the first step, comparable to that of the popular ferroelectric material PbTiO_3_ (ref. 27).

In the second stage, we examine how the application of a perpendicular electric field reduces the kinetic barrier by lifting the degeneracy of the two polarized states. Our detailed calculations show that the activation barrier associated with the three-step concerted mechanism decreases linearly with the electric displacement field in the range of the field strength less than 0.3 V Å^−1^ (Fig. 3a). It is worthwhile to note that the electric field induces much more dramatic variation in the energy difference between the two oppositely polarized states than in the activation barrier. As shown in Fig. 3a, an electric displacement field of 0.3 V Å^−1^ gives rise to an energy difference as large as 0.056 eV per unit cell, which is expected to result in a nearly ten times population difference of the two oppositely polarized states at room temperature. Moreover, for a ferroelectric domain of a practical device, the energy difference between the two polarized states is proportional to the domain size. Therefore, the population difference increases exponentially with the domain size. Although the activation barrier is also proportional to the domain size, given the relatively small activation barrier of 0.066 eV per unit cell, it is still possible to have an optimal domain size that gives rise to not only a sufficiently large energy difference to drive the reversal of the electric polarization but also a moderate activation barrier to make the kinetic process accessible at room temperature. Experimentally, an electric displacement field as large as 0.3 V Å^−1^ had been demonstrated previously, for example, to open a sizable band gap in bilayer graphene^28^, which is expected to have a lower electric breakdown voltage than the present systems. In addition, it is important to point out that the reversal of the out-of-plane electric polarization accompanies with the reversal of the in-plane electric polarization for the FE-ZB′ phase, as illustrated in Fig. 3b. We also examine the effects of the application of an in-plane electric field on the kinetics of the electric polarization reversal process. The calculated results, as summarized in Fig. 3b, indicate that an in-plane electric field of 0.03 V Å^−1^ applied in the [110] direction gives rise to an energy difference as large as 0.142 eV per unit cell between the two oppositely polarized states, which is expected to result in a more than 200 times population difference of the two oppositely polarized states at room temperature. These features may offer an alternative approach to switch the orientation of the out-of-plane polarization by the application of an in-plane electric field.

**Figure 4:**
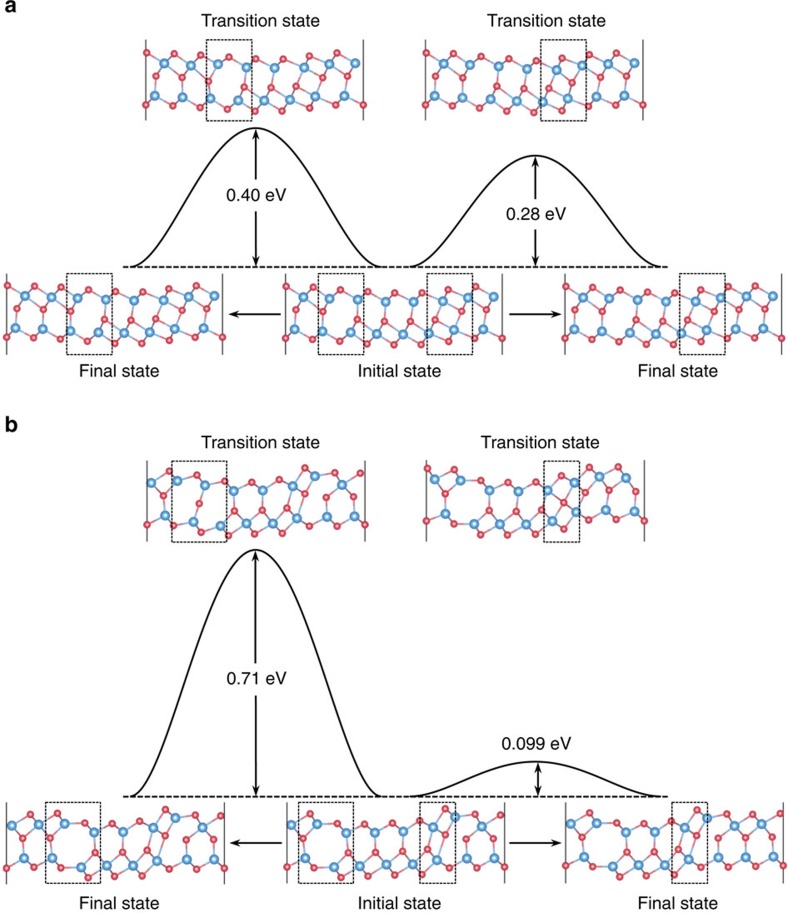
Kinetic pathways of domain wall motion. Initial states containing four possible domain wall structures between two ferroelectric domains of one quintuple layer In_2_Se_3_ in the FE-ZB′ phase with opposite electric polarizations are shown in **a**,**b** (at the bottom centre), along with the energy profiles, final states (at the bottom left and right), and transition states (at the top) of the kinetic pathways involved in the motion of the domain walls. The black dashed squares indicate the positions of the domain walls.

So far we have limited ourselves to ideal 2D systems of infinite size. In physically realistic growth conditions, the systems are more likely to contain different types of defects, especially domain walls^3^,^5^. Here we show that the electric polarization reversal process can be further facilitated by the presence of a domain wall between two oppositely polarized domains. In doing so, we construct four possible domain wall structures, as shown in the initial states of Fig. 4a,b, by moving half of the Se atoms in the central layer initially aligned to one In layer to the neighbouring sites aligned to the other In layer. The total formation energy of the two domain walls in the configuration as shown in Fig. 4a (0.22 eV per unit cell) is much lower than that in Fig. 4b (1.45 eV per unit cell). The calculated activation barriers in the low-energy configuration are 0.40 and 0.28 eV per unit cell along the domain wall, respectively, much lower than the barrier of 0.85 eV per unit cell against the direct shifting mechanism of the central Se layer discussed earlier.

### In_2_Se_3_-based van der Waals heterostructures

**Figure 5:**
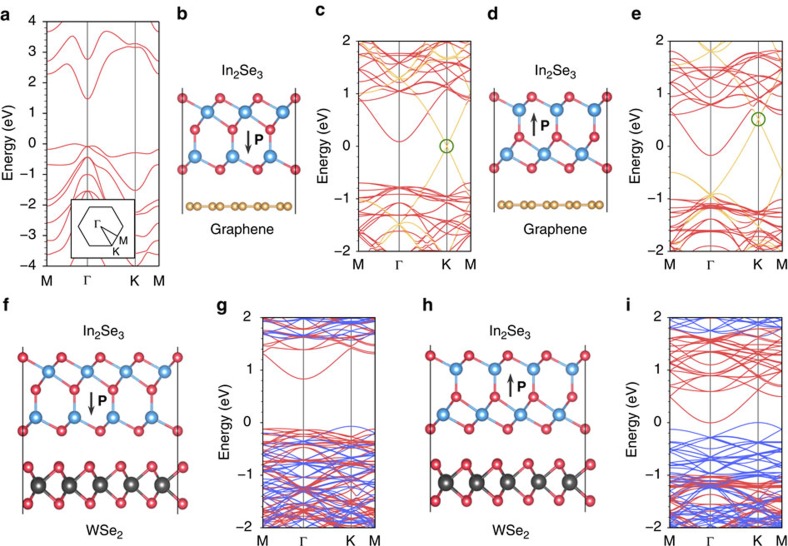
Electronic structures of In_2_Se_3_-based heterostructures. (**a**) Electronic band structure of one quintuple layer (QL) ferroelectric In_2_Se_3_ in the FE-ZB′ phase (calculated by HSE06); here the inset shows the first Brillouin zone with the high symmetric points of Γ, Μ and ϰ indicated. (**b**–**e**) Demonstration of a tunable Schottky barrier at the interface of a one QL FE-ZB′ In_2_Se_3_/graphene heterostructure. (**b**,**d**) The side views of the heterostructure. The corresponding electronic band structures are shown in **c**,**e** (calculated by GGA-PBE). The bands derived from the In_2_Se_3_ layer and the graphene layer are highlighted in red and yellow, respectively. The green circles indicate the Dirac points of the graphene layer. (**f**–**i**) Demonstration of a significant band gap reduction in a one QL FE-ZB′ In_2_Se_3_/WSe_2_ heterostructure. (**f**,**h**) The side views of the heterostructure with the electric dipole of the In_2_Se_3_ layer pointing downwards and upwards, respectively. The corresponding electronic band structures are shown in **g**,**i** (calculated by GGA-PBE). The bands derived from the In_2_Se_3_ layer and the WSe_2_ layer are highlighted in red and blue, respectively. The Fermi level of each system is shifted to energy zero in all the band structure plots.

Next, we demonstrate the device potential of the discovered 2D ferroelectric materials in van der Waals heterostructures, focusing on the electrical transport properties. As a reference, a single ferroelectric In_2_Se_3_ QL is a semiconductor with an indirect band gap of 1.46 eV (calculated by HSE06, 0.78 eV by GGA-PBE) (Fig. 5a). Owing to the presence of the out-of-plane electric polarization of the ferroelectric layer, there is a built-in electric field within the material, leading to different alignments of the energy bands with respect to the vacuum level on different sides of a given ferroelectric QL. For a ferroelectric In_2_Se_3_ QL, such a difference is as large as 1.37 eV (calculated by HSE06). As a van der Waals 2D material is stacked with a ferroelectric In_2_Se_3_ layer, the energy bands of the two components are approximately aligned with respect to the vacuum level, due to their weak van der Waals interaction. Therefore, as different sides of the ferroelectric layer are in contact with the other 2D material, different band alignments result in different global electronic structures. As the first specific system, we consider a bilayer heterostructure by stacking a QL of ferroelectric In_2_Se_3_ onto a single-layer graphene, which is a non-ferroelectric semimetal. As shown in Fig. 5b–e, the Schottky barrier across the interface can be altered by switching the electric dipole orientation of the In_2_Se_3_ layer. The magnitude of the electric dipoles of the system is 0.11 and 0.03 eÅ per In_2_Se_3_ unit cell for the two oppositely polarized configurations as shown in Fig. 5b,d, respectively. The next bilayer heterostructure system considered is formed by stacking a QL of ferroelectric In_2_Se_3_ on a monolayer of WSe_2_, which is a non-ferroelectric semiconductor. As shown in Fig. 5f–i, the band shift leads to a significant band gap reduction when switching the electric dipole orientation of the In_2_Se_3_ layer. The magnitude of the electric dipoles of the system is 0.10 and 0.06 eÅ per In_2_Se_3_ unit cell for the two oppositely polarized configurations as shown in Fig. 5f,h, respectively. For both heterostructures, the reduction of the electric dipoles in one of the polarized configurations can be attributed to the screening effects due to the charge transfer between the two layers as indicated in Fig. 5e,i. The influence of the graphene and WSe_2_ layers on the energetics and kinetics of the polarization reversal processes of the ferroelectric In_2_Se_3_ layer is discussed in Supplementary Note 3. The observed tunable band alignments with the ferroelectric layer can be exploited for different technological applications, such as for non-volatile memory devices or in graphene-based electronics. It is particularly worthwhile to note that the tunability in the properties can be achieved by the application of an external field, but the desired functionalities can be preserved even after the external field is removed. To provide a generic guideline for the design of desirable heterostructures, a schematic diagram of the band alignments of a single ferroelectric In_2_Se_3_ QL is provided in Supplementary Fig. 13.

### Family of 2D ferroelectric III_2_–VI_3_ compounds

So far we have limited our discussions on the intrinsic ferroelectric properties of In_2_Se_3_. Next, we show that ferroelectric 2D van der Waals materials can be harboured in a wider family of the III_2_–VI_3_ materials. Our DFT calculations suggest that the ferroelectric phases, FE-ZB′ and FE-WZ′, are also the ground states of Al_2_S_3_, Al_2_Se_3_, Al_2_Te_3_, Ga_2_S_3_, Ga_2_Se_3_, Ga_2_Te_3_, In_2_S_3_ and In_2_Te_3_ when such materials are prepared in the QL form. Their semiconducting electronic band structures and optimal lattice constants are shown in Supplementary Fig. 14, and their dynamic stability is confirmed by the lack of imaginary phonon modes in the calculated phonon band structures (Supplementary Fig. 15). We further note that all the In-containing compounds have both the stable ferroelectric (FE-ZB′ and FE-WZ′) and metastable fcc′ structures, while all the Ga-containing compounds only possess the stable ferroelectric structures, with the fcc-derived structure being unstable.

## Conclusions

In this work, we have discovered a class of stable single-layer van der Waals 2D ferroelectric materials rooted in III_2_–VI_3_ compounds that possess both intrinsic out-of-plane and in-plane electric polarization, which can be reversed through readily accessible kinetic pathways with the assistance of a modest out-of-plane or in-plane electric field. In a broader prospective, these discoveries add an important branch to the family tree of 2D materials. Proper integration of these materials with other classes of 2D systems is expected to substantially broaden the tunability and device potential of van der Waals heterostructures.

## Methods

### Computational methods

The first-principles DFT calculations were performed using the Vienna *Ab Initio* Simulation Package^29^. Valence electrons were described using the projector-augmented wave^30^,^31^ method. The plane wave expansions were determined by the default energy cutoffs given by the Vienna *Ab Initio* Simulation Package projector-augmented wave potentials. The exchange and correlation functional was treated using the PBE^32^ parametrization of GGA for structural relaxations and total energy calculations. For the band structure calculations of pristine III_2_–VI_3_ compounds, we also used the hybrid functional of Hyed-Scuseria-Ernzerhof (HSE06) (ref. 33). To model the 2D films, the supercells contain a unit cell of single QL structures with a vacuum region of more than 15 Å. A saw-like self-consistent dipole layer was placed in the middle of the vacuum region to adjust the misalignment between the vacuum levels on the different sides of the film due to the intrinsic electric polarization. A Γ-centred 12 × 12 × 1 Monkhorst-Pack^34^ *k*-mesh was used for *k*-point sampling. Optimized atomic structures were achieved when forces on all the atoms were <0.005 eV A^−1^. The in-plane electric polarization was evaluated by using the Berry phase method^35^. The climbing image nudged elastic band method^36^ is used to determine the energy barriers of the various kinetic processes. In the heterostructure calculations, we included the van der Waals corrections as parameterized in the semiempirical DFT-D3 method^37^. More details are provided in Supplementary Note 1.

### Data availability

All relevant data are available from the authors.

## Additional information

**How to cite this article:** Ding, W. *et al*. Prediction of intrinsic two-dimensional ferroelectrics in In_2_Se_3_ and other III_2_-VI_3_ van der Waals materials. *Nat. Commun.* **8,** 14956 doi: 10.1038/ncomms14956 (2017).

**Publisher's note**: Springer Nature remains neutral with regard to jurisdictional claims in published maps and institutional affiliations.

## Supplementary Material

Supplementary Information Supplementary Figures, Supplementary Notes and Supplementary References

Peer Review File
