# Supplementary material for: Prediction of intrinsic two-dimensional ferroelectrics in In2Se3 and other III2-VI3 van der Waals materials
Source: Nat Commun. 2017 Apr 7;8:14956. doi: 10.1038/ncomms14956 (PMC5385629; doi:10.1038/ncomms14956)
Supplement: Supplementary Information — Supplementary Figures, Supplementary Notes and Supplementary References [file ncomms14956-s1.pdf]

Supplementary Note 1: Computational details

First-principles calculations

Our first-principles calculations were performed using the Vienna *Ab Initio* Simulation Package (VASP)[1], which is based on density functional theory and plane wave basis sets with the projector-augmented wave (PAW) method[2,3]. The exchange and correlation functional was treated using the Perdew-Burke-Ernzerhof (PBE)[4] parameterization of generalized gradient approximation (GGA). For pristine III_2-VI_3 quintuple layers (QLs), in addition to PBE, we also used the hybrid functional of Hyed-Scuseria-Ernzerhof (HSE06)[5] to calculate their electronic band structures. The supercells contain a unit cell of the two-dimensional III_2-VI_3 quintuple layer with a vacuum region of more than 15 Å to separate the two-dimensional layer from their images of the neighboring supercells in the vertical direction due to the periodic condition. A Γ-centered 12×12×1 Monkhorst-Pack[6] *k*-point mesh was used for Brillion zone sampling. The outmost *s* and *p* electrons for Al, S, Se, and Te, *s*, *p*, and *d* electrons for Ga and In, *s* and *d* electrons for W, and *s* and *p* electrons for C are treated as valence states in the PAW potentials. The energy cut-offs of the plane wave basis sets for each material investigated were set as follows: 260 eV for Al_2S_3, 240 eV for Al_2Se_3, 240 eV for Al_2Te_3, 300 eV for Ga_2S_3, 300 eV for Ga_2Se_3, 300 eV for Ga_2Te_3, 260 eV for In_2S_3, 250 eV for In_2Se_3, 240 eV for In_2Te_3, 250 eV for In_2Se_3/WSe_2, 400 eV for In_2Se_3/graphene. A self-consistent dipole layer was placed in the middle of the vacuum region to balance the difference of the vacuum levels on the different sides of the two-dimensional layer, due to the intrinsic electric polarization. Electronic minimization was performed with a tolerance of 10^{-6} eV, and ionic relaxation was performed with a force tolerance of 0.005 eV $Å^{-1}$ on each ion. All these parameters were carefully tested to ensure the convergence and accuracy. The climbing image nudged elastic band method[7] was used to determine the energy barriers of the various kinetic processes.

Structure searching

We performed a thorough search for the most stable structure of 1 QL In_2Se_3, guided by the experimental observations. Each atomic layer in a QL contains only one element, either In or Se,

with the atoms arranged in an equilateral triangular lattice and initially placed on one of the three sublattice sites, A, B, or C, as illustrated in Fig. 1b of the main text. In addition to the commonly accepted stacking sequence of the elements, Se-In-Se-In-Se, we also considered all the different stacking sequences of 1 QL In_2Se_3, including two symmetric sequences (i.e., Se-In-Se-In-Se and In-Se-Se-Se-In) and four asymmetric sequences (i.e., In-In-Se-Se-Se, In-Se-In-Se-Se, In-Se-Se-In-Se, and Se-In-In-Se-Se). For each sequence, we have further considered all possible stacking configurations, including 18 inequivalent configurations for each symmetric case (as shown in Supplementary Fig. 1) and 30 inequivalent configurations for each asymmetric case (as shown in Supplementary Fig. 3). In addition, we also considered the fcc' structure for each sequence. All the supercells were built on 1×1 equilateral triangular lattices with the shape fixed and the in-plane lattice constant fully relaxed. Our DFT calculations confirmed that the ferroelectric phases FE-ZB' and FE-WZ' in the stacking sequence of Se-In-Se-In-Se are the most stable and degenerate structures. The structures in the other sequences all have total energies at least 1.2 eV higher than the most stable ferroelectric structures.

Phonon calculations

The phonon band structures were calculated using the finite displacement method. The interatomic forces were computed with a 6×6×1 supercell and a 2×2×1 *k*-point mesh using the VASP code. The tolerance for the force convergence used for the phonon calculations was 10^{-8} eV $Å^{-1}$. The force-constant matrices and phonon frequencies were calculated using the PHONOPY code[8]. We note that in some of the calculated phonon band structures small imaginary acoustic phonon modes appear in the vicinity of the gamma point, which are very likely to be caused by the numerical inaccuracy due to the limited supercell size. Even if the small imaginary phonon modes do exist in a realistic freestanding system, they are all located in the vicinity of the gamma point, and are of acoustic nature, corresponding to a collective vibration mode with a long wavelength approaching infinity. Such a long-wavelength instability may induce ripples in the structure, but is not expected to significantly affect the overall structural stability and ferroelectricity of the materials.

Molecular dynamics

We performed *ab initio* molecular dynamics (MD) implemented in VASP to verify the stability of 1 QL of the ferroelectric FE-ZB' In_2Se_3. The canonical NVT (N: number of particles, V: volume, T: temperature) ensemble was used with Nosé thermostat[9]. A 4×4×1 supercell and a 2×2×1 *k*-point mesh were used. Each time step is set to 2 fs, and simulations were conducted for more than 5000 steps (10 ps) at 400K. To consider the effects of volume, simulations with different lattice constants, 4.106 Å for the most stable FE-ZB' structure and 4.048 Å for the metastable fcc' structure, were used, respectively.

Out-of-plane electric polarization

The out-of-plane electric polarization for 2D systems is well defined by the classical electrodynamics due to the presence of a vacuum region, and it can be calculated by direct integrating ρ times z over the whole supercell, where ρ is the local charge density and z is the coordinate in the out-of-plane axis. The external electric field in the out-of-plane direction was applied by inserting a dipole layer in the middle of the vacuum region, and the magnitude of the dipole layer was self-consistently determined by making the electric field in the vacuum region away from the slab be a preset value during the calculations. In the vacuum, the electric field **E** is equal to the electric displacement field **D**, and the **D** in the vacuum is equal to the **D** in the ferroelectric layer, according the electrodynamics. Thus, the magnitude of the external electric field set in the calculations is equal to the **D** in the ferroelectric layer. For a standard experimental setup where the external electric field is applied through a dielectric layer[10], the electric field **E** in the dielectric layer is not equal to the **E** in the ferroelectric layer and the difference depends on the dielectric constant of the dielectric layer, but the displacement field **D** in the dielectric layer is always equal to the **D** in the ferroelectric layer, which is independent of the dielectric constant of the dielectric layer. Therefore, we use **D** instead of **E** to characterize the external electric field. Practically, the magnitude of **D** is determined by the magnitude of the applied voltage between the metal electrodes, the thickness of the dielectric layer, and its dielectric constant [10].

In-plane electric polarization

The evaluation of the in-plane electric polarization was based on the modern theory of polarization using the Berry phase method[11], as implemented in the VASP code, with both electronic and ionic contributions being taken into account. We chose the fcc structure, which is centrosymmetric, as the non-polar reference. The transformation of the FE-ZB' and FE-WZ' to the fcc structure is illustrated in Supplementary Fig. 10a. The evolution of the in-plane electric polarization was mapped out along a continuous adiabatic insulating pathway connecting the non-polar fcc structure and the ferroelectric phase, on which the position of the structure in the supercell was adjusted to ensure the continuity of the total electric polarization, as shown in Supplementary Fig. 10b for the FE-ZB' case. The external in-plane electric field was applied by imposing a finite homogeneous electric field using the PEAD (perturbation expression after discretization) approach[12], which corresponds to the electric field determined experimentally according to the applied voltage and the distance between two electrodes. The evolution of the energy profile along the most effective kinetic pathway as illustrated in Fig. 2b for the FE-ZB' structure to reverse the electric polarization at an applied in-plane external electric field up to 0.03 V $Å^{-1}$ is shown in Supplementary Fig. 10c. The magnitude of the in-plane electric polarization along the most effective kinetic pathway for the FE-ZB' structure is shown in Supplementary Fig. 10d.

Kinetics of domain wall motion

To investigate the kinetics of the electric polarization reversal process via the motion of domain walls in a single ferroelectric In_2Se_3 QL, we construct a large supercell (8×1) that contains oppositely polarized domains. The domain walls are formed by shifting half of the central-layer Se atoms in an ideal single-domain ferroelectric In_2Se_3 QL to neighboring positions that reverse the local electric polarization. As illustrated in Supplementary Fig. 11, there are two inequivalent ways of shifting the central-layer Se atoms to form domain structures, corresponding to the angle formed between the moving direction of the central-layer Se atoms and the domain wall orientation to be either 30° (Supplementary Fig. 11a) or 90° (Supplementary

Fig. 11b). For each case, two different domain wall structures are formed within a supercell, the kinetic processes of the domain wall motions for the four possible domain wall structures are investigated as shown in Fig. 4 of the main text. The motions of the domain walls are mainly via the moving of the central-layer Se atoms, with the rest atoms fully relaxed.

Van der Waals heterostructures

In the In_2Se_3/WSe_2 and In_2Se_3/graphene heterostructure calculations, we use large supercells to match the two components. According to the optimal in-plane lattice constants of one monolayer of In_2Se_3 (4.106 Å), WSe_2 (3.325 Å), and graphene (2.468 Å) calculated by GGA-PBE, we choose a supercell containing a 4×4 In_2Se_3 layer and a 5×5 WSe_2 layer for the In_2Se_3/WSe_2 case, corresponding to a mismatch of 1.2%, and a supercell containing a 3×3 In_2Se_3 layer and a 5×5 graphene layer for the In_2Se_3/graphene case, corresponding to a mismatch of 0.2%. The in-plane lattice parameters of the supercells are set to the lattice constant of In_2Se_3, and the lattices of WSe_2 and graphene are adjusted to match the In_2Se_3 layer accordingly. The van der Waals corrections as parameterized in the semi-empirical DFT-D3 method[13] are included, and default parameters by the VASP code are used (VDW_S6 = 1.0000; VDW_S8 = 0.7220; VDW_SR = 1.2170; rs18 = 1.0000; alpha6 = 14.0000; alpha8 = 16.0000; k1-k3 = 16.0000 1.3333 -4.0000; VDW_RADIUS = 50.2022 Å; VDW_CNRADIUS = 21.1671 Å). The most stable stacking geometries are obtained by optimizing the structures from different initial configurations.

Interlayer binding energy in In_2Se_3 multilayers

Our DFT calculations for bilayer systems with van der Waals corrections (DFT-D3) show that the binding energy between ferroelectric In_2Se_3 layers is similar to those of transition metal dichalcogenides calculated within the same computational scheme. For MoS_2, $MoSe_2$, WS_2, and WSe_2, the calculated interlayer binding energies are 0.235, 0.272, 0.243, and 0.292 eV per unit cell, respectively, and the calculated interlayer binding energy of an In_2Se_3 bilayer in the ferroelectric FE-ZB' phase is 0.281 eV per unit cell. When comparing the area averaged interlayer binding energies, the calculated value for In_2Se_3 is 0.019 eV $Å^{-2}$, close to the value of

0.017 eV Å^{-2} for graphene. Therefore, In_2Se_3 should be able and have been shown to be separable into monolayers via mechanical exfoliation or other separation methods, similar to graphene and transition metal dichalcogenides.

Depolarizing field effects and critical thickness

The calculations to examine the effect of the depolarizing field induced by metal electrodes on the stability of the ferroelectric phase of In_2Se_3 were performed for supercells containing 1 QL In_2Se_3 sandwiched between two graphite electrodes in short-circuit[14], as illustrated in Supplementary Figs. 7a and 7b for the In_2Se_3 layer in the ferroelectric FE-ZB' phase and non-polar fcc' phase, respectively. Similar to the In_2Se_3/graphene heterostructure calculations, a 3×3 In_2Se_3 layer was used to match with a 5×5 graphite multilayer containing 5 atomic layers. The periodic boundary conditions along the out-of-plane direction (*c* axis) naturally impose the required short-circuit condition between the electrodes. A 5×5×2 *k*-point mesh with the Γ point was used. The van der Waals corrections as parameterized in the semi-empirical DFT-D3 method[13] were included. The length of *c* axis was optimized, and all atoms were fully relaxed. Since the FE-ZB' phase and fcc' phase have different in-plane lattice parameters, to compare their total energies in the presence of graphite electrodes, we used the same in-plane lattice constant for the two cases and then made the following corrections. For example, we set the in-plane lattice constant to that of the FE-ZB' phase, and corrected the total energy of the fcc' phase as $E = E_0 - E_{IS\text{-}ZB'\text{-}vdW} + E_{IS\text{-}fcc'}$, where E_0 is the calculated total energy of a QL of fcc' In_2Se_3 with the graphite electrodes, $E_{IS\text{-}ZB'\text{-}vdW}$ is the energy of a freestanding fcc' In_2Se_3 layer in the FE-ZB' lattice with van der Waals corrections, and $E_{IS\text{-}fcc'}$ is the energy of a freestanding fcc' In_2Se_3 layer in the fcc' lattice without van der Waals corrections. Here the fictitious modifications within the In_2Se_3 layer because of the van der Waals corrections were also considered. For the calculations with the in-plane lattice constant set to the fcc' lattice, the total energy of the FE-ZB' case was also corrected in a similar way. The calculated results indicate that the ferroelectric FE-ZB' phase is still more stable than the non-polar fcc' phase even with the depolarizing field. The energy differences are 0.047 eV and 0.039 eV per In_2Se_3 unit cell for the

cases of the in-plane lattice constant set to the FE-ZB’ lattice and fcc’ lattice, respectively. Supplementary Fig. 7c shows in-plane averaged electrostatic potential plotted along the *c* axis for the FE-ZB’ case. The different potentials at the two outmost Se layers of In_2Se_3 also suggest that the electric polarization induced built-in electric field is still preserved in the presence of the graphite electrodes.

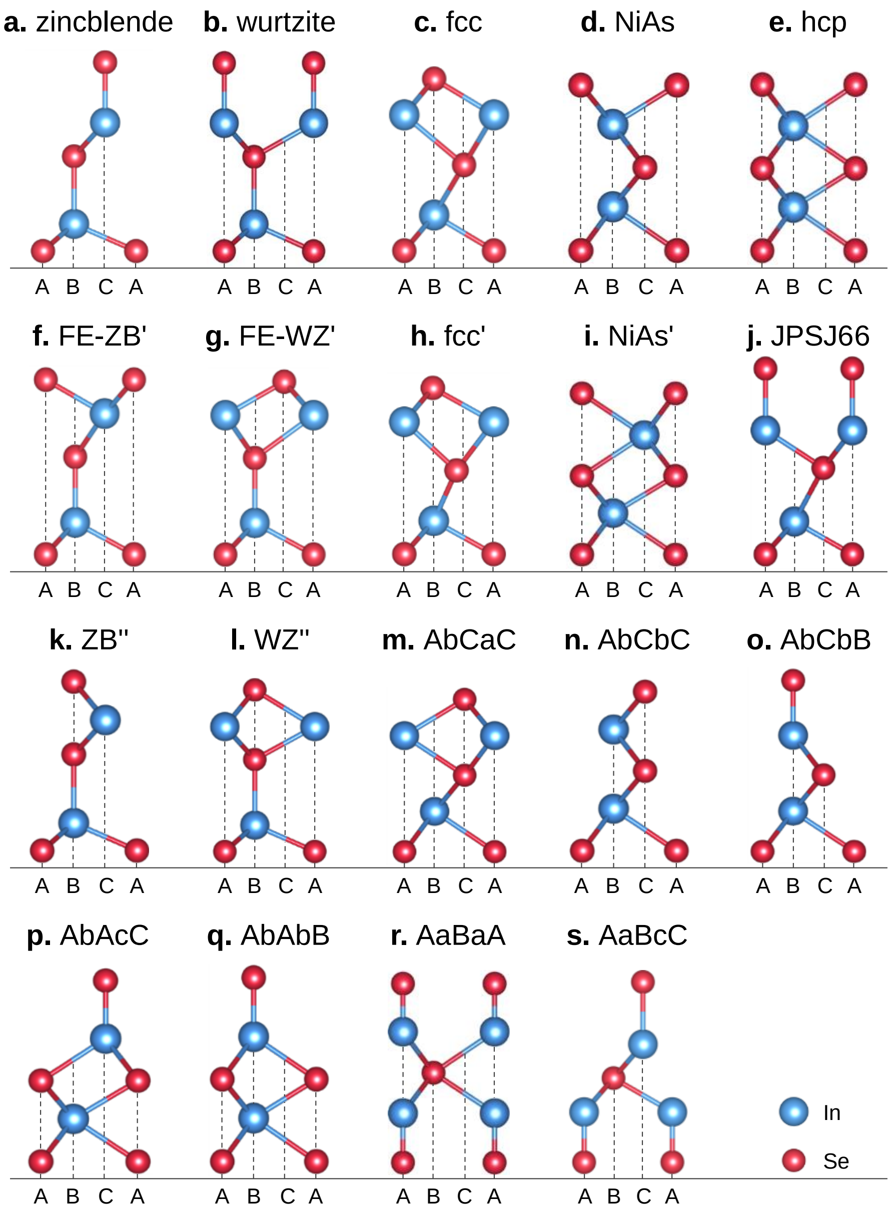

Supplementary Figure 1: Side views of all structures examined for 1 quintuple layer In_2Se_3 in the symmetric stacking sequence of Se-In-Se-In-Se. (**a-s**) The name for each structure is provided at the top of each pane. All the other configurations considered with a different symmetric stacking sequence can be obtained by proper elemental replacement. In all the structures, each atomic layer in a QL contains only one elemental species with the atoms arranged in a triangular lattice occupying A, B, or C sites as illustrated in Fig. 1b.

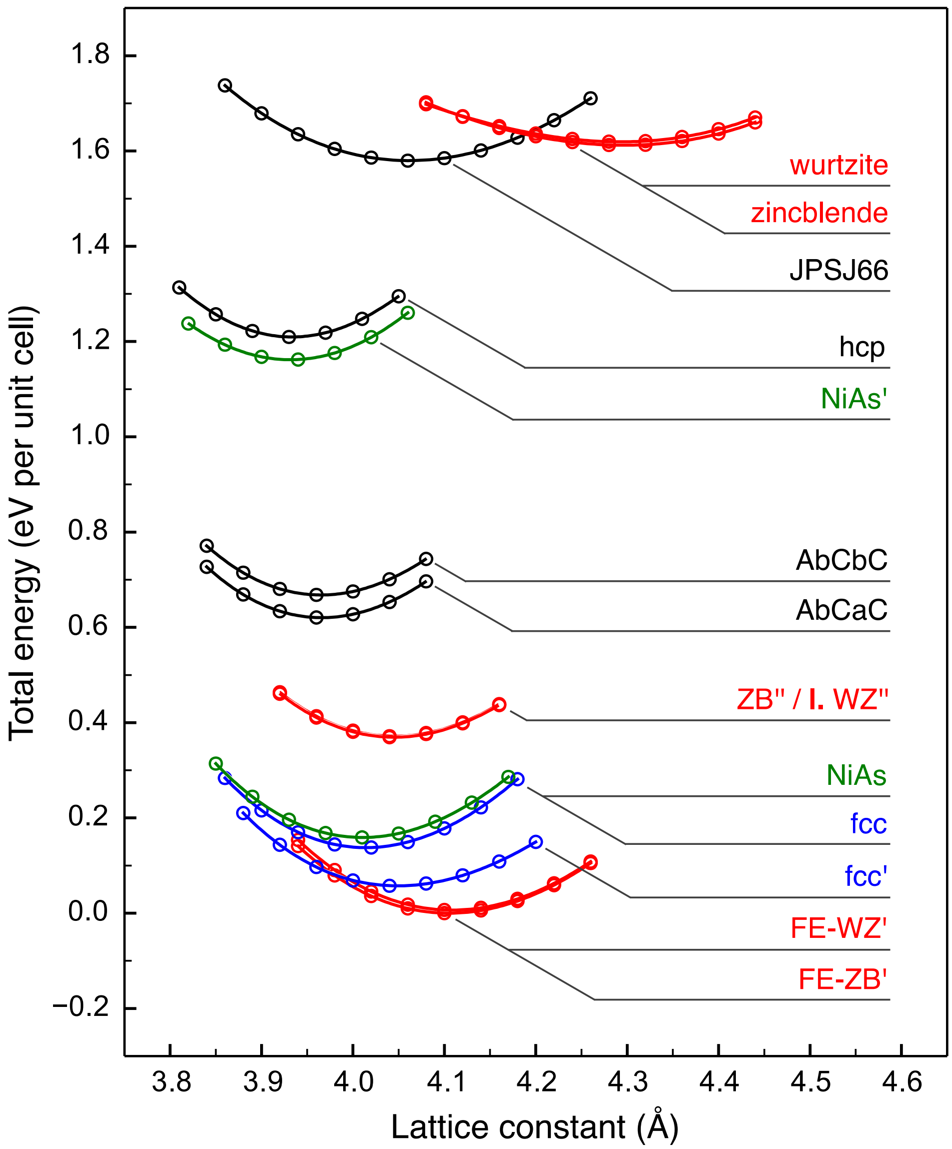

Supplementary Figure 2: Calculated total energy versus lattice constant for the structures of 1 quintuple layer In_2Se_3 enumerated in Supplementary Fig. 1. The structures listed in Supplementary Fig. 1 but not presented in this figure have much higher energies. The structures derived from zincblende or wurtzite structure are coded in red, the ones from fcc structure in blue, and the ones from NiAs structure in green. The two energetically degenerate ground state structures, FE-ZB' and FE-WZ', correspond to the α phase, and the second most stable structure, fcc', corresponds to the β phase.

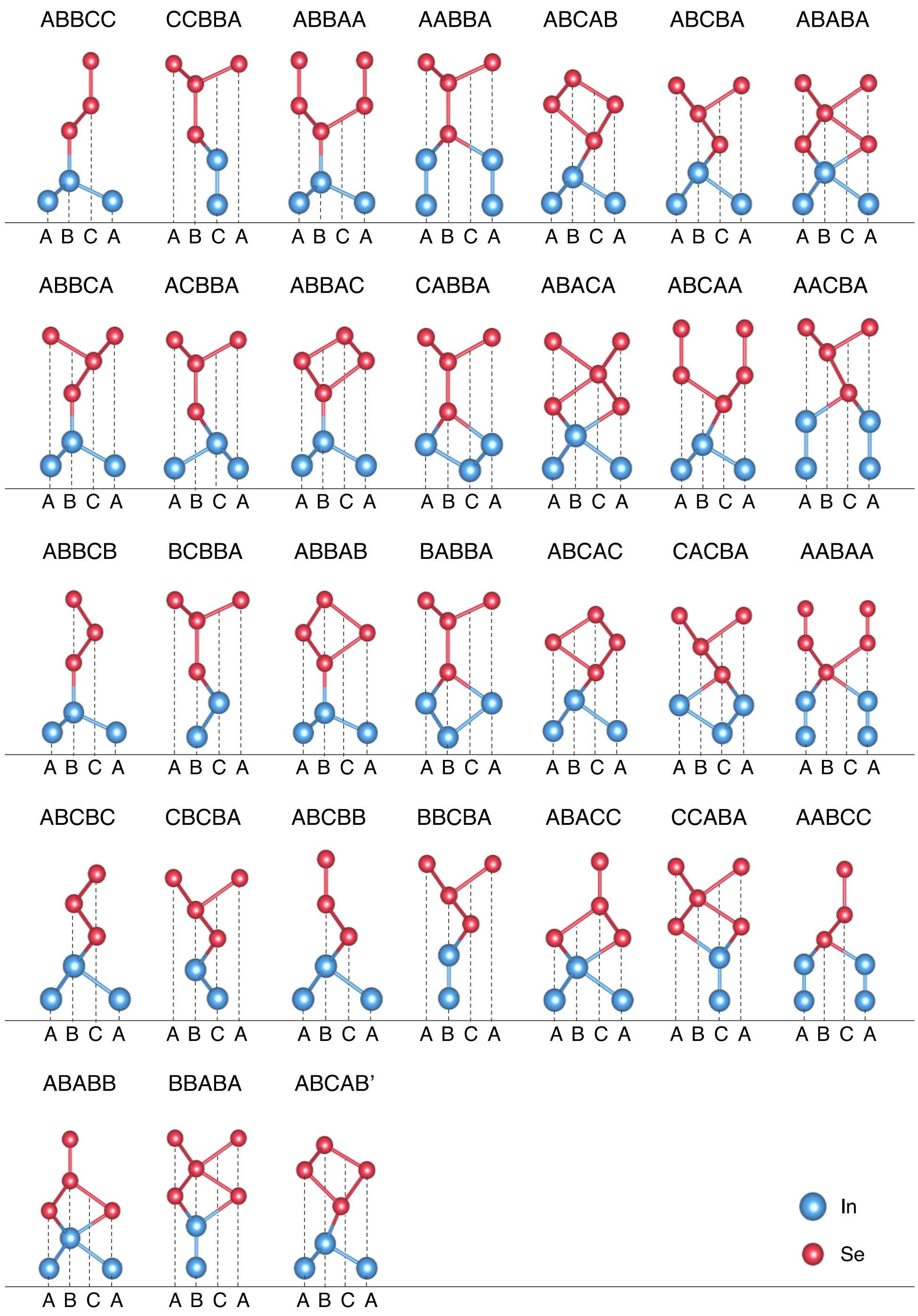

Supplementary Figure 3: All structures examined for 1 quintuple layer In_2Se_3, corresponding to the asymmetric stacking sequence of In-In-Se-Se-Se. All the other configurations considered with a different asymmetric stacking sequence can be obtained by proper elemental replacement.

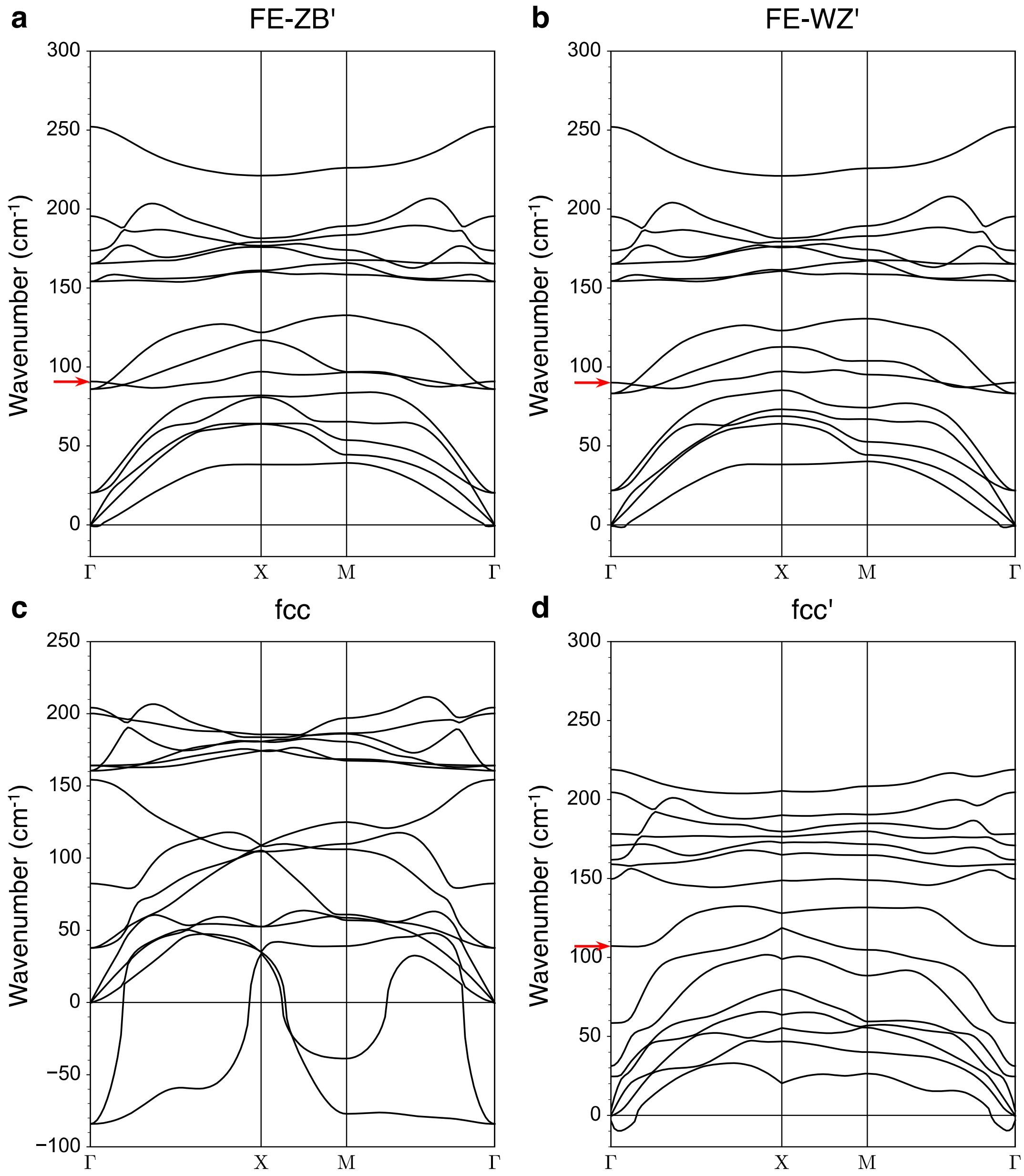

Supplementary Figure 4: Calculated phonon band structures. (**a**) Ferroelectric FE-ZB’ structure. (**b**) Ferroelectric FE-WZ’ structure. (**c**) fcc structure. (**d**) fcc’ structure. The positions of the Raman active A1 mode are indicated by the red arrows for the FE-ZB’, FE-WZ’, and fcc’ structures, respectively.

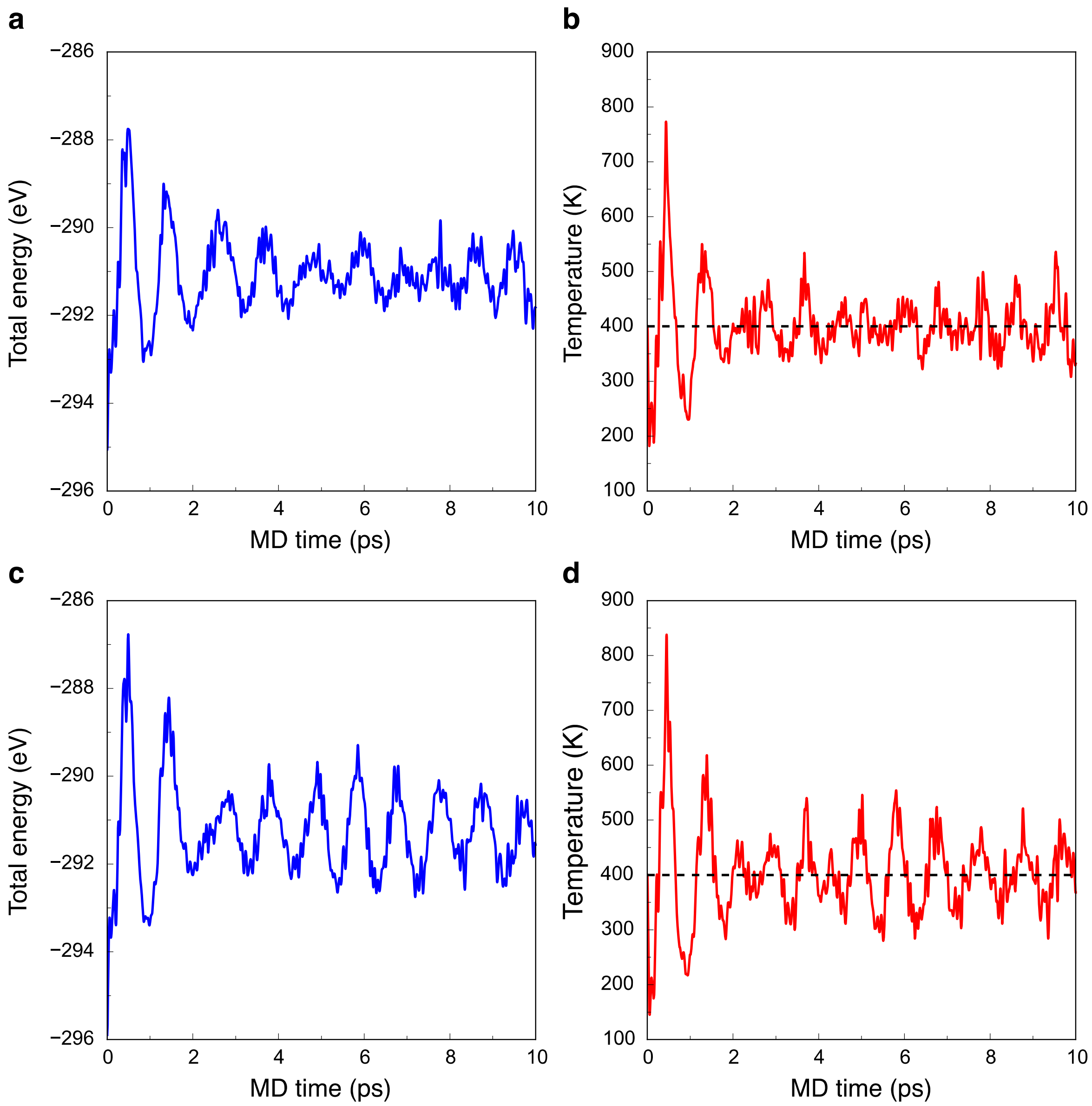

Supplementary Figure 5: *Ab initio* MD simulations of 1 quintuple layer FE-ZB' In_2Se_3 at 400K. Simulations with different lattice constants were performed. (**a** and **b**) The lattice constant is set to that of the FE-ZB' phase (4.106 Å). (**c** and **d**) The lattice constant is set to that of the fcc' phase (4.048 Å). (**a** and **c**) The evolution of the total internal energy during the MD simulations. (**b** and **d**) The evolution of temperature during the MD simulations. The results indicate that the structures were still maintained at the initial FE-ZB' phase during the MD simulations for 10 ps.

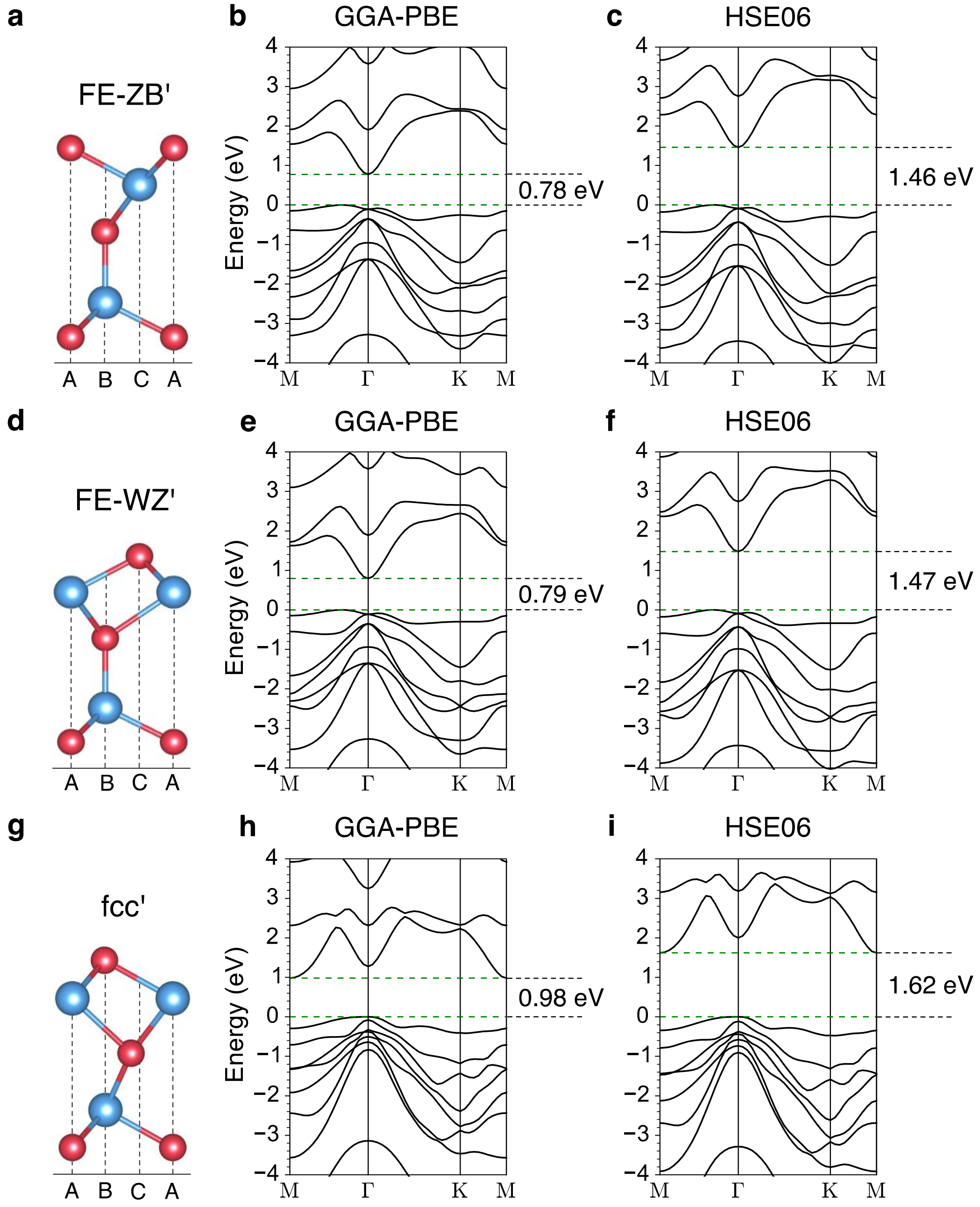

Supplementary Figure 6: Calculated electronic band structures of 1 quintuple layer In_2Se_3. (**a**, **d**, and **g**) Side views of FE-ZB' phase (**a**), FE-WZ' phase (**d**), and fcc' phase (**g**). The band structures shown in **b**, **e**, and **h** were calculated by GGA-PBE, and **c**, **f**, and **i** by HSE06.

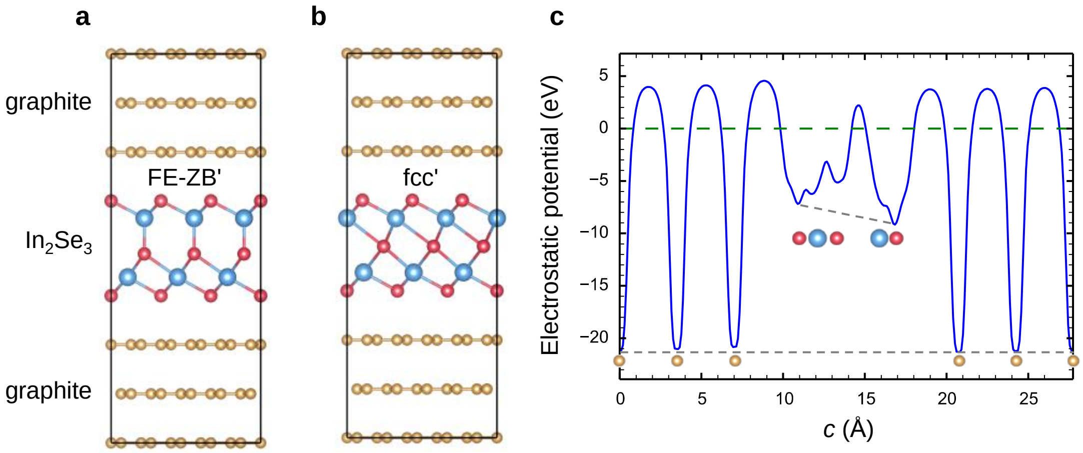

Supplementary Figure 7: 1 quintuple layer In_2Se_3 sandwiched between two graphite electrodes in short-circuit. (**a** and **b**) Side views of supercells with the In_2Se_3 layer in FE-ZB' phase and fcc' phase, respectively. (**c**) In-plane averaged electrostatic potential for the FE-ZB' case. Each pit in the potential plot corresponds to the position of an atomic layer. The balls below the pits in yellow, blue, and red represent C, In, and Se layers, respectively. The grey dashed line connecting the potentials between the two outmost Se layers with a pronounced slope indicates that the electric polarization induced built-in electric field is still preserved in the presence of the graphite electrodes.

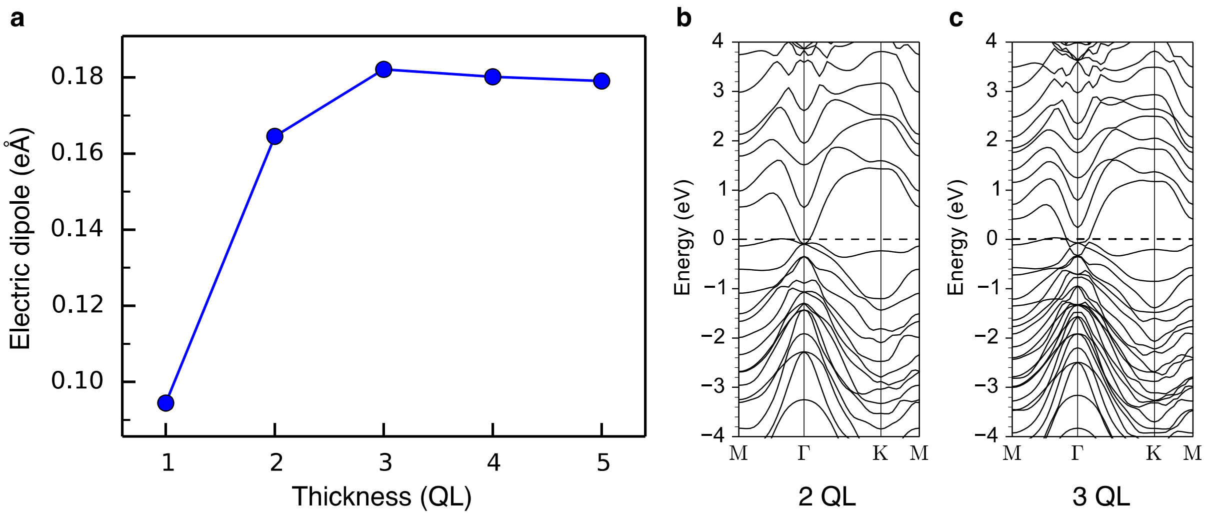

Supplementary Figure 8: Electric polarization of multilayer In_2Se_3 films. (**a**) Electric dipole of a ferroelectric In_2Se_3 film as a function of the film thickness. Electric dipoles in all the QLs are initiated and stabilized in the same orientation. (**b** and **c**) Band structures of a 2 QL and 3 QL film calculated by GGA-PBE, respectively.

Supplementary Note 2: The magnitude of the electric dipole saturates as the thickness is above 2 QLs. The band structure of 2 QL film indicates that the band gap of the system is closed, because the energy bands of the ferroelectric QLs shift relatively to each other as their polarization is aligned in the same orientation, due to the built-in electric field of ferroelectricity. Above 2 QLs, further increase of the thickness will not further significantly enlarge the polarization of the system, because the overlaying of the conduction band and valence band leads to charge transfer from one side to the other side of the film, giving rise to an opposite electric field to balance the built-in electric field of the ferroelectric multilayers and hindering the further increase of the polarization. It is worth noting that, energetically, except for the 2 QL case, the polarization of all the ferroelectric QLs in thicker multilayers prefers to be aligned in the same orientation as dictated by the classical electrodynamics. For the 2 QL case, the electric dipoles of the two QLs slightly prefer to be aligned in opposite orientations (by 0.005 eV per formula unit), both pointing outward, resulting in a net zero polarization.

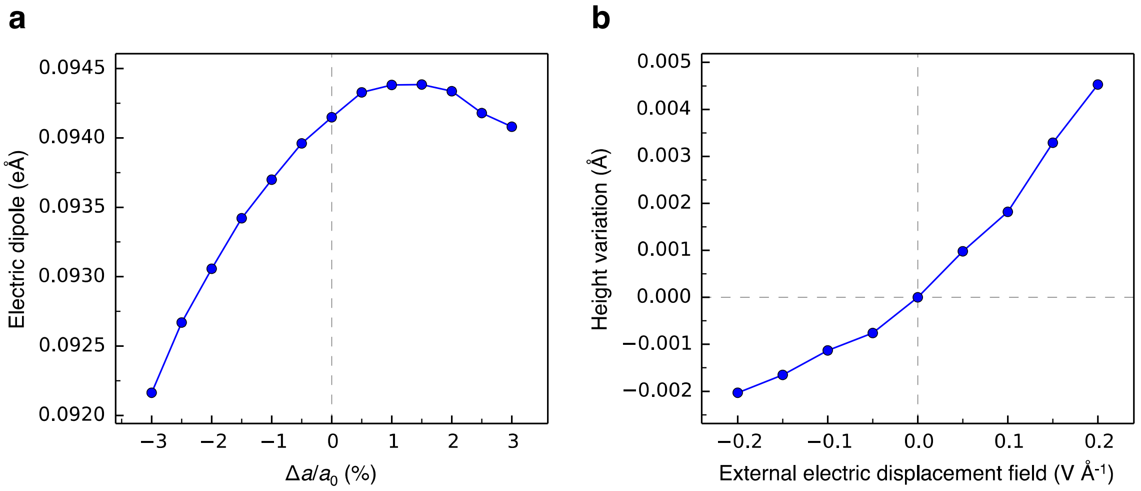

Supplementary Figure 9: Piezoelectric properties of 1 quintuple layer (QL) **In_2Se_3 in the FE-ZB' phase calculated by GGA-PBE.** (**a**) The magnitude of the out-of-plane electric dipole as a function of the in-plane lattice deformation. a_0 represents the equilibrium in-plane lattice constant. (**b**) The height variation of a 1 QL In_2Se_3 film as a function of the external electric field applied in the vertical direction.

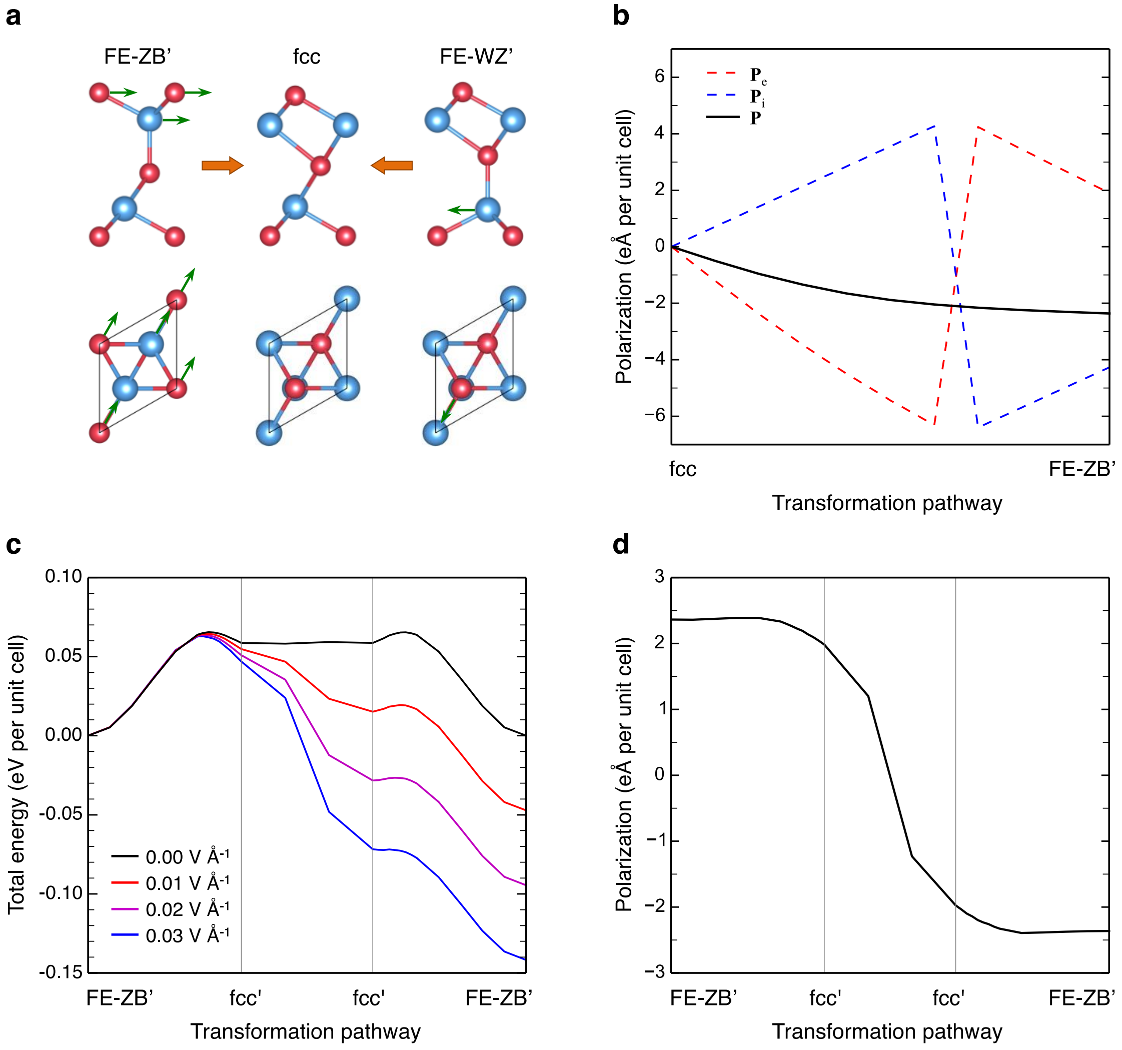

Supplementary Figure 10: In-plane electric polarization. (**a**) Schematic illustration of the transformation pathways from the FE-ZB' and FE-WZ' structures to the fcc structure. The green arrows attached to atoms indicate the directions of atomic motion during the structural transformation. (**b**) The evolution of the electronic contribution $\mathbf{P_e}$ (the red dashed line), the ionic contribution $\mathbf{P_i}$ (the blue dashed line), and the total in-plane electric polarization **P** (the black solid line) along the adiabatic pathway from the fcc structure to the FE-ZB' structure. (**c**) The evolution of the energy profile along the most effective kinetic pathway as illustrated in Fig. 2b for the FE-ZB' structure to reverse the electric polarization at an applied in-plane external electric field up to 0.03 V Å^{-1}. (**d**) The magnitude of the in-plane electric polarization along the most effective kinetic pathway for the FE-ZB' structure.

a

b

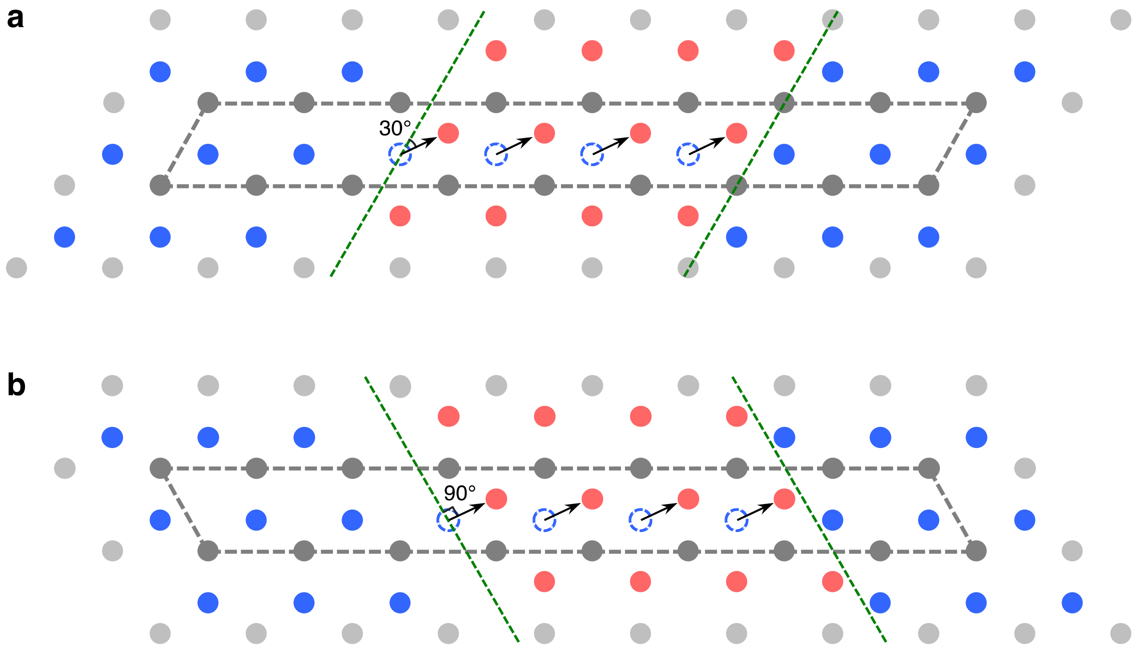

Supplementary Figure 11: Top views of two supercells used in the domain wall calculations. The grey dashed lines indicate the supercells. Both blue and red dots denote the positions of the central-layer Se atoms, while different colors represent different domains. The blue circles denote the initial positions of the central-layer Se atoms of the red domain before the shift, as indicated by the arrows. In **a**, the direction of the shift of the central-layer Se atoms forms an angle of 30° with the domain wall. In **b**, the direction of the shift of the central-layer Se atoms is perpendicular to the domain wall.

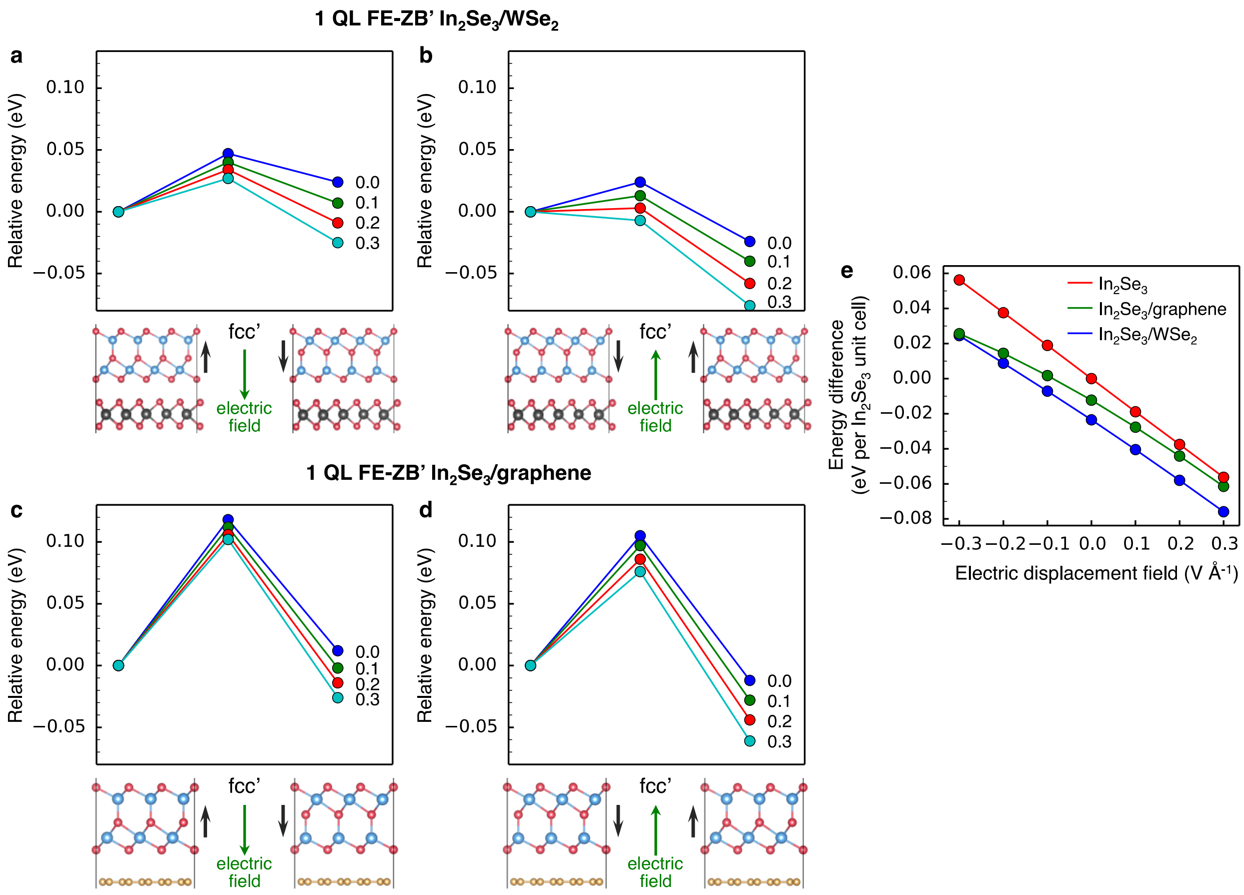

Supplementary Figure 12: Evolution of the total energies of the In_2Se_3-based heterostructures with the variation of an external electric displacement field applied in the vertical direction. (**a** and **b**) For In_2Se_3/WSe_2; (**c** and **d**) For In_2Se_3/graphene. In **a-d**, the unit of the electric displacement field is in V Å^{-1}. **e**, the energy difference between the two polarized states as a function of the electric displacement field for a freestanding In_2Se_3 QL, the In_2Se_3/WSe_2, and In_2Se_3/graphene heterostructures.

Supplementary Note 3: The results, as shown in Supplementary Fig. 12, indicate that without the electric field, the presence of WSe_2 or graphene lifts the degeneracy of the two oppositely polarized states of In_2Se_3, and results in an energy difference of 0.024 eV and 0.012 eV for the WSe_2 and graphene case, respectively. Consequently, switching from one dipole orientation is expected to become easier but from the other orientation become harder. The application of an external electric field can readily diminish such energy differences, as shown in Supplementary

Fig. 12, and the resultant fields needed to reverse the dipole directions in the heterosystems are expected to be slightly larger in magnitude than that in the freestanding case. Furthermore, from Supplementary Fig. 12e, it can be seen that the slopes of the energy differences between the two polarized states as a function of the electric field for both heterostructures are close to that of a freestanding ferroelectric In_2Se_3 QL, suggesting that the extra screening effects of the WSe_2 and graphene layer are still rather weak, likely due to their ultrathin nature.

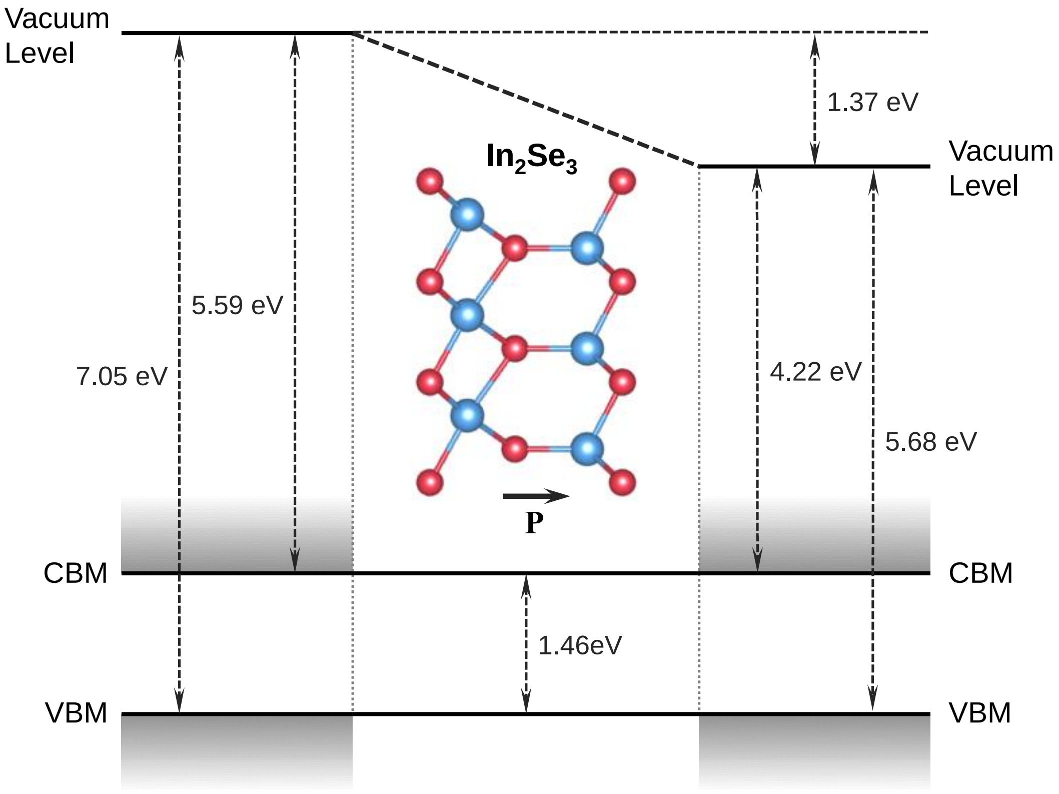

Supplementary Figure 13: Schematic illustration of the band offsets of a ferroelectric In_2Se_3 quintuple layer. The band offsets of the conduction band minimum (CBM) and valence band maximum (VBM) of 1 QL In_2Se_3 in the FE-ZB' phase with respect to the respective vacuum levels on the two surfaces of the film. The difference in the alignments of the energy bands with respect to the vacuum level on different surfaces is due to the presence of an out-of-plane electric polarization induced built-in electric field within the film. For a ferroelectric In_2Se_3 QL, the difference is as large as 1.37 eV as calculated by HSE06.

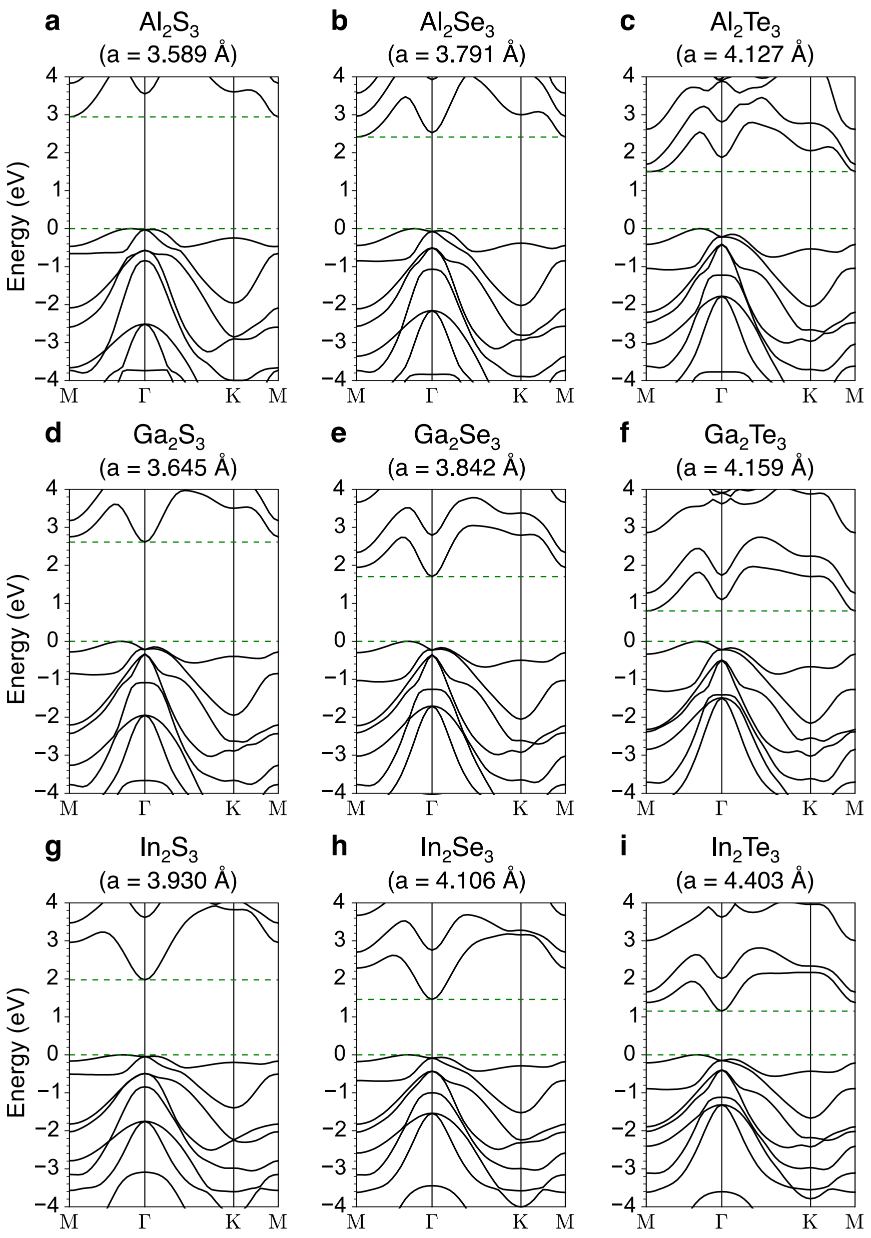

Supplementary Figure 14: Calculated electronic band structures of 1 quintuple layer III_2-VI_3 compounds in the ferroelectric FE-ZB' phase. (**a-i**) The name of each compound is provided at the top of each pane. The in-plane lattice constants of all the compounds are fully relaxed with the optimal values provided above the band structure panels. All the band structures are calculated by HSE06.

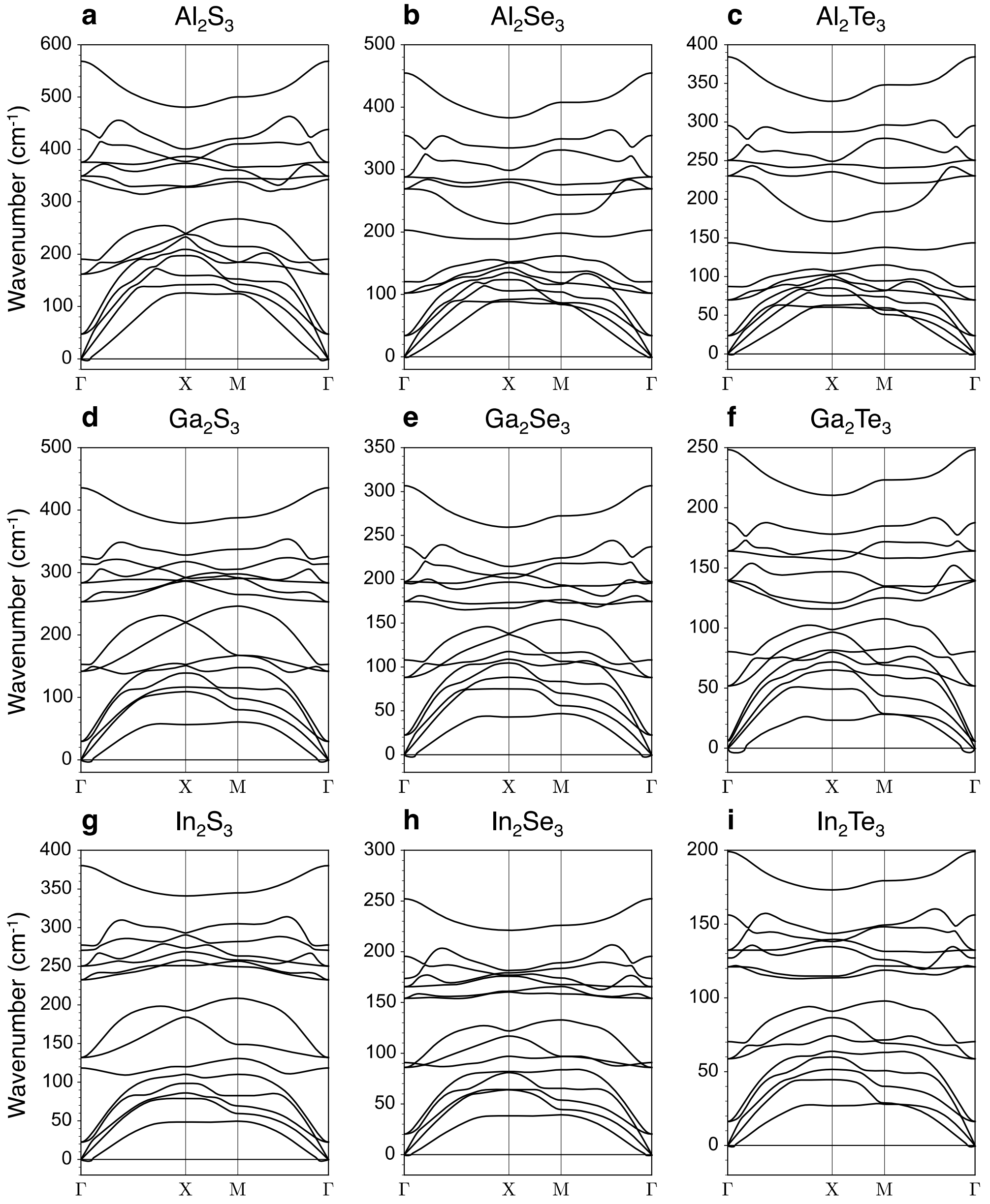

Supplementary Figure 15: Calculated phonon band structures of 1 quintuple layer III_2-VI_3 compounds in the ferroelectric FE-ZB' phase. (**a-i**) The name of each compound is provided at the top of each pane. The absence of pronounced imaginary phonon mode indicates that all these compounds are dynamically stable, if they can be grown into the quintuple layer form.

Supplementary References

1. Kresse, G. & Furthmüller, J. Efficient iterative schemes for *ab initio* total-energy calculations using a plane-wave basis set. *Phys. Rev. B* **54,** 11169-11186 (1996).
2. Blöchl, P. E. Projector augmented-wave method. *Phys. Rev. B* **50,** 17953-17979 (1994).
3. Kresse, G. & Joubert, D. From ultrasoft pseudopotentials to the projector augmented-wave method. *Phys. Rev. B* **59,** 1758-1775 (1999).
4. Perdew, J. P., Burke, K. & Ernzerhof, M. Generalized gradient approximation made simple. *Phys. Rev. Lett.* **77,** 3865-3868 (1996).
5. Krukau, A. V., Vydrov, O. A., Izmaylov, A. F. & Gustavo, E. S. Influence of the exchange screening parameter on the performance of screened hybrid functionals. *J. Chem. Phys.* **125,** 224106 (2006).
6. Methfessel, M. & Paxton, A. T. High-precision sampling for Brillouin-zone integration in metals. *Phys. Rev. B* **40,** 3616-3621 (1989).
7. Henkelman, G., Uberuaga, B. P. & Jónsson, H. A climbing image nudged elastic band method for finding saddle points and minimum energy paths. *J. Chem. Phys.* **113,** 9901-9904 (2000).
8. Togo, A. & Tanaka, I. First principles phonon calculations in materials science. *Scr. Mater.* **108,** 1-5 (2015).
9. Nosé, S. A unified formulation of the constant temperature molecular dynamics methods. *J. Chem. Phys.* **81,** 511-519 (1984).
10. Zhang, Y. *et al*. Direct observation of a widely tunable bandgap in bilayer graphene. *Nature* **459,** 820-823 (2009).
11. King-Smith, R. D. & Vanderbilt, D. Theory of polarization of crystalline solids. *Phys. Rev. B* **47,** 1651-1654 (1993).

12. Nunes, R. W. & Gonze, X. Berry-phase treatment of the homogeneous electric field perturbation in insulators. *Phys. Rev. B* **63,** 155107 (2001).

13. Grimme, S., Antony, J., Ehrlich, S. & Krieg, H. A consistent and accurate *ab initio* parametrization of density functional dispersion correction (DFT-D) for the 94 elements H-Pu. *J. Chem. Phys.* **132,** 154104 (2010).

14. Junquera, J. & Ghosez, P. Critical thickness for ferroelectricity in perovskite ultrathin films. *Nature* **422,** 506-509 (2003).
