# Supplementary material for: Prediction of intrinsic two-dimensional ferroelectrics in In2Se3 and other III2-VI3 van der Waals materials
Source: Nat Commun. 2017 Apr 7;8:14956. doi: 10.1038/ncomms14956 (PMC5385629; doi:10.1038/ncomms14956)
Supplement: Peer Review File [file ncomms14956-s2.pdf]

Editorial Note: this manuscript has been previously reviewed at another journal that is not operating a transparent peer review scheme. This document only contains reviewer comments and rebuttal letters for versions considered at Nature Communications.

Reviewers' Comments:

Reviewer #1 (Remarks to the Author)

The authors have sufficiently addressed my questions, and the manuscript is acceptable for publication.

Reviewer #3 (Remarks to the Author)

The authors have carefully replied my questions raised in the last two rounds and revised the manuscript. Although I still question if their structure is truly the ground state, I understand that the authors have tried their best to theoretically search for stable structures. Therefore, I recommend it to be published in Nature Communications.
